# Spatio-temporal variation of the endangered Dupont’s Lark diet across Iberia and Morocco

**DOI:** 10.1371/journal.pone.0301318

**Published:** 2024-12-11

**Authors:** Julia Zurdo, Daniel Bustillo-de la Rosa, Adrián Barrero, Julia Gómez-Catasús, Margarita Reverter, Cristian Pérez-Granados, Jesús T. García, Javier Viñuela, Julio C. Domínguez, Manuel B. Morales, Juan Traba

**Affiliations:** 1 Department of Ecology, Terrestrial Ecology Group, Universidad Autónoma de Madrid (TEG-UAM), Madrid, Spain; 2 Centro de Investigación en Biodiversidad y Cambio Global, Universidad Autónoma de Madrid (CIBC-UAM), Madrid, Spain; 3 Ecology Department, Universidad de Alicante, Alicante, Spain; 4 Instituto de Investigación en Recursos Cinegéticos (IREC, CSIC-UCLM-JCCM), Ciudad Real, Spain; 5 Pyrenean Institute of Ecology (IPE, CSIC), Jaca, Spain; East Carolina University, UNITED STATES OF AMERICA

## Abstract

A species’ diet is highly dependent on the availability of food resources in space and time, as well as on intrinsic factors such as sex or age. Accurate assessments of variations in the diet composition of bird populations across spatial scales, seasons and demographic groups are essential not only for understanding the basic ecology of species, but also for the conservation of endangered ones. However, our current knowledge about how birds’ diet change according to spatio-temporal variations or intrinsic factors is very limited. Here, we used a multi-marker metabarcoding approach to characterize the diet of a declining shrub-steppe passerine, the Dupont’s Lark (*Chersophilus duponti*), throughout a large part of its global distribution range. We also investigated spatial, phenological and sexual variations in its diet. Using markers from two genomic regions (18S and COI), we analyzed fecal samples from 303 adult Dupont’s larks from Morocco and Spain during the breeding and non-breeding seasons. Overall, arthropods from the orders Coleoptera, Lepidoptera, Julida and Orthoptera were the main prey consumed by Dupont’s Larks. We found that Dupont’s Lark diet varied spatially, as well as temporally, reflecting dietary plasticity in response to changes in prey availability across landscapes and the species’ phenological periods. High dietary overlap and no differences between sexes were observed, suggesting similar foraging behavior and nutritional requirements in both sexes. This is the first study providing detailed information on Dupont’s Lark food ecology over much of its distribution, which is fundamental for the management and conservation of this declining steppe species.

## Introduction

The study of animals’ diet is central to understand the survival and maintenance of individuals and populations [1], as diet influences many dimensions of animals’ life history, including physiology, habitat use, migration, survival and breeding success [2]. However, accurately characterizing animal diets is challenging, because of the effort required to collect precise data and the variations in food resources used by animals, which can be affected by both abiotic and biotic factors [3,4].

The diet of a species can change over time and space owing to variations in availability of food resources [5]. Arthropods, for instance, vary in abundance throughout the year, and they typically have an uneven spatial distribution, even at local scale (i.e., within habitats [6]). Consequently, seasonal, annual, and regional variations may occur in the diet of insectivorous animals [2,7,8]. Accordingly, several insectivorous animals exhibit dietary plasticity, which is a key factor that enables species to deal with environmental changes affecting prey distribution and abundance [9]. According to optimal foraging theory, the diet of generalist predators is expected to vary seasonally, shifting between alternative prey species depending on which are more abundant [10]. It is therefore essential to investigate dietary variation, at both spatial and temporal scales, to fully understand the food ecology of animals, which is, in turn, critical for wildlife management and the conservation of endangered species [11]. Such knowledge can help identify when these species are more vulnerable or when their populations are most likely to experience food limitations [12].

Diet can also vary among individuals of the same population due to intrinsic factors, such as sex, reproductive status or age [8,13,14]. Indeed, sex-related differences on birds’ diet have been typically attributed to morphological and behavioral differences [15,16], but also to differing nutritional demands during the breeding season due to distinct reproductive roles [17]. Sexual dietary differentiation is expected to decrease intraspecific competition for food resources [18], which is especially important when resource availability is limited [19]. Altogether, an accurate knowledge of dietary variation at both species-specific and spatio-temporal levels might be crucial in identifying the underlying causes of decline of endangered species.

**Fig 1:**
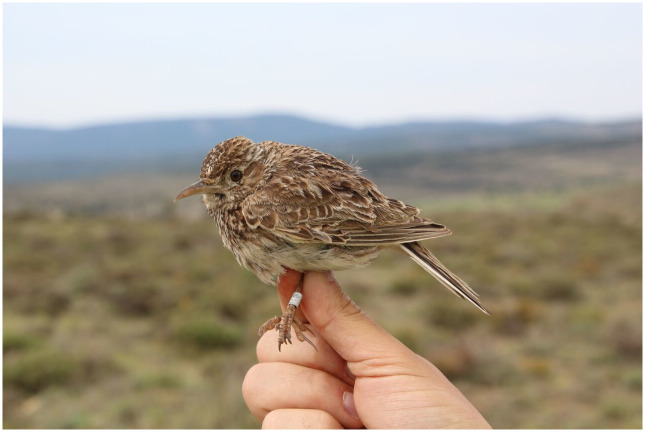
Cover photograph. A male Dupont’s Lark (*Chersophilus duponti*). Photographed by Adrián Barrero–TEG-UAM.

Dupont’s Lark (*Chersophilus duponti*) (Fig 1) is a scarce steppe passerine of the Alaudidae family, whose distribution is restricted to Spain in Europe, and to the Maghreb (Morocco, Algeria and Tunisia), Libya and Egypt in Africa [20]. Dupont’s Lark is listed as “Vulnerable” at a European level [21], and in Spain the species has recently been listed as “Endangered” [22]. This species is strongly habitat-selective, occurring exclusively in flat (slope less than 15%), treeless shrub-steppe systems [23], which makes it highly vulnerable to habitat alterations. In fact, the land-use changes (agricultural intensification, abandonment of traditional sheep grazing, afforestation, infrastructure development) and habitat fragmentation that drive the regression and degradation of steppes in Spain and the Maghreb [20,24–26] affect the entire passerine community which depend on this particular habitat [27], and are leading to the loss and isolation of Dupont’s Lark populations [28]. The degradation of steppe habitat also causes decreases in arthropod availability [29], a critical threat to this primarily insectivorous species [30,31]. Indeed, the European Dupont’s Lark population showed a decrease of 29.9% from 2004 to 2022 [32]. In Morocco, the species has also experienced a population decline and a reduction of its breeding area [20]. Population sizes and trends in the rest of its African distribution are less well known, but a recent study suggests regressive trends in the Maghreb, at least in Tunisia [26].

Knowledge on Dupont’s Lark diet is limited and restricted to specific Spanish populations. [31] described the species as insectivore and occasionally granivore, which was supported by a study based on visual examination of four gizzard contents collected in early autumn [33]. The diet of nestlings of this species was studied by [34], who noted the importance of Orthoptera, Lepidoptera, Araneae and Coleoptera. Recent studies using DNA metabarcoding have provided the first detailed information on adult and nestling diet of this species [14,30,35], but the information in these studies is limited to data collected from a single study area in Central Spain during the breeding season. These local studies revealed that Dupont’s Lark most frequently consumed Coleoptera, Lycosidae (Araneae), Julidae (Julida), Orthoptera, Cydnidae (Hemiptera) and Lepidoptera [14], showing age-related dietary differences [14]. However, there are currently no studies assessing how the diet of this steppe-specialist species varies over large spatial scales and across different periods of the species’ phenology.

In this study, we used multi-marker DNA metabarcoding to conduct the first comprehensive diet analysis of Dupont’s Lark over most of the range of the nominal *C*. *d*. *duponti* subspecies [20]. DNA metabarcoding is a molecular technique that overcomes the limitations of traditional methods (e.g., time-consuming, coarse taxonomic resolution, lack of identification of prey items that leave no hard remains [36,37]), providing the ability to identify a wider range of ingested taxa with higher taxonomic resolution [38]. In addition, the integration of dietary data from different markers in a multi-marker approach overcomes problems of primer specificity and bias [37]. Here, we aimed to provide an accurate description of dietary composition across spatial scales, phenology and demographic groups, and to explore whether the diet of Dupont’s Lark varies and what factors determine such variation. We predicted that the composition of Dupont’s Lark diet will vary at both macro-spatial (between countries) and country-level scales (within Spain), likely reflecting geographical changes in prey availability influenced by differences in landscape characteristics. We also expected diet variation at different periods of the species’ phenology due to differences in arthropod availability along the year. For instance, we expected a greater consumption of phytophagous arthropods (e.g., Orthoptera, Heteroptera) during the non-breeding period, as the availability of this group increases during this period in autumn in the Iberian shrub-steppes [39]. Finally, we predicted sexual differences in diet composition, probably reflecting different nutritional demands of both sexes during the breeding season. An expected difference is, for instance, that females will consume calcium-rich prey (e.g., millipedes of the order Julida) more frequently than males, since they need to meet the nutritional requirements of egg-laying. The findings from this study will be useful to inform Dupont’s Lark conservation strategies across its distribution range to ensure the survival of this species, potentially benefiting other declining steppe passerines.

## Materials and methods

### Study sites

**Fig 2:**
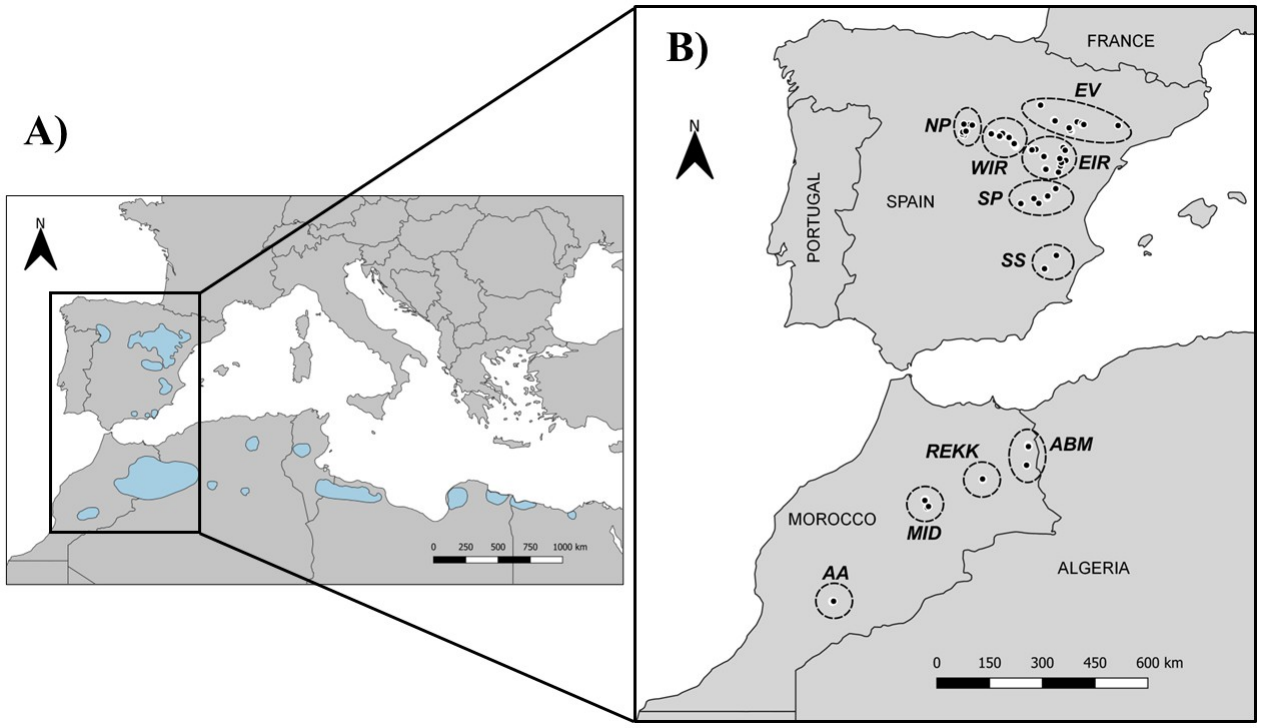
Location of the study area. (A) Location of the study area within the Dupont’s Lark global distribution (blue polygons) based on [21]. The subspecies *Chersophilus duponti duponti* is distributed in the Iberian Peninsula, Morocco, northern Algeria and Tunisia; the subspecies *C*. *duponti margaritae* is distributed in southern Algeria and Tunisia, Libya and Egypt. (B) Spanish and Moroccan sampling points (black dots). Fieldwork regions are indicated with dashed lines: Northern Plateau (NP), Ebro Valley (EV), Western Iberian Range (WIR), Eastern Iberian Range (EIR), Southern Plateau (SP), and Southern Spain (SS) in Spain; Aïn Bni Mathar (ABM), Plateau of Rekkam (REKK), Midelt-Missour (MID), and Anti-Atlas (AA) in Morocco. © EuroGeographics for the administrative boundaries.

Fieldwork was carried out in two countries, Spain and Morocco, covering a large part of the species’ distribution range [40] (Fig 2).

In Spain, sampling was conducted in spring, during the Dupont’s Lark breeding seasons (March-June) of 2017–2019 and in autumn, during the non-breeding season (September-November) of 2017. We collected fecal samples in six geographic regions throughout the range based on their ecological and geographic characteristics [32,41] (Table 1, Fig 2): Northern Plateau (NP), Ebro Valley (EV), Western Iberian Range (WIR), Eastern Iberian Range (EIR), Southern Plateau (SP), and Southern Spain (SS). These regions differ mainly in the amount of habitat suitable to Dupont’s Lark and their degree of fragmentation [42]. WIR, EIR and EV regions are the main core areas of Dupont’s Lark, where the largest patches of continuous typical habitat of the species in Spain are found, whereas the rest are isolated and highly fragmented areas [41]. The dominant landscape, in all regions, is flat steppes covered by low scrub and a high proportion of bare ground [23,40].

**Table 1:** Number of Dupont’s Lark successful samples after molecular analysis from Morocco and Spain and across study regions of Spain, broken down by sex, species’ phenology, and year.

Country	Total	Sex	Phenology (Year)
**Morocco**	28	Male = 27	Breeding season = 27 (2020)
			Non-breeding season = 0
		Female = 1	Breeding season = 1 (2020)
			Non-breeding season = 0
**Spain**	171	Male = 159	Breeding season = 148 (2017 = 49, 2018 = 77, 2019 = 22)
			Non-breeding season = 11 (2017)
		Female = 12	Breeding season = 12 (2017 = 6, 2018 = 4, 2019 = 2)
			Non-breeding season = 0
***Spanish Region***			
**Ebro Valley**	30	Male = 29	Breeding season = 29 (2018 = 27, 2019 = 2)
			Non-breeding season = 0
		Female = 1	Breeding season = 1 (2018)
			Non-breeding season = 0
**Northern Plateau**	14	Male = 12	Breeding season = 12 (2018 = 8, 2019 = 4)
			Non-breeding season = 0
		Female = 2	Breeding season = 2 (2018)
			Non-breeding season = 0
**Western Iberian Range**	85	Male = 77	Breeding season = 66 (2017 = 49, 2018 = 12, 2019 = 5)
			Non-breeding season = 11 (2017)
		Female = 8	Breeding season = 8 (2017 = 6, 2018 = 1, 2019 = 1)
			Non-breeding season = 0
**Eastern Iberian Range**	25	Male = 25	Breeding season = 25 (2018)
			Non-breeding season = 0
		Female = 0	Breeding season = 0
			Non-breeding season = 0
**Southern Plateau**	15	Male = 14	Breeding season = 14 (2018 = 3, 2019 = 11)
			Non-breeding season = 0
		Female = 1	Breeding season = 1 (2019)
			Non-breeding season = 0
**Southern Spain**	2	Male = 2	Breeding season = 2 (2018)
			Non-breeding season = 0
		Female = 0	Breeding season = 0
			Non-breeding season = 0

In Morocco, we sampled in late winter/early spring (February-March) of 2020, coinciding with the breeding season of larks in such area. We collected samples throughout the range, in the four geographic regions determined by [20] as suitable sites for the species: Aïn Bni Mathar (ABM) in northeastern Morocco, Plateau of Rekkam (REKK) and Midelt-Missour region (MID) at the central plateau, and the Anti-Atlas region (AA) in southwestern Morocco (Fig 2). Further visualization of the Moroccan sample locations can be found in Fig I in S1 Appendix of the Supporting Information. Steppe vegetation was dominated by alfa grass *Macrochloa tennacissima* except in the southwestern (AA), where *Artemisia* spp. was the dominant species [20]. Despite collecting samples from different geographic regions, Morocco was treated as a single entity in subsequent statistical analyses due to the small sample size of most regions (see Table I in S1 Appendix).

### Sample collection

For collecting Dupont’s Lark fecal samples (*n* = 303), we first captured adult individuals using spring-traps baited with mealworms (*Tenebrio molitor*) and a species-specific recording to attract them, which is a male-biased sampling [43]. Individuals were ringed to avoid pseudoreplication and released at the site of capture. Sex was determined through wing length following [44], who determined that adult males had wing lengths > 97 mm while females had wing lengths < 97 mm. Active brood patch in females during the breeding season was also a sex discriminant trait. We collected fresh fecal samples from the ground at the spring-trap during the capture or produced in the ringing bags. To prevent contamination, in Spain the ringing bags were washed with bleach before each use, whereas in Morocco sterile filter paper placed inside the bags was used. Samples were stored in individual 1.5-mL plastic vial tubes with 98% ethanol and were frozen (-20 ºC) at the end of the field day until they were processed in the laboratory. All birds were captured and handled in accordance with both national and international guidelines and under permits from Moroccan and Spanish authorities. All procedures were approved by l’Agence Nationale des Eaux et Forêts (ref: 01/2020) and the Local Ethical Committee for Animal Experiments of the Universidad Autónoma de Madrid (CEI80-1468-A229).

### Inclusivity in global research

Additional information regarding the ethical, cultural, and scientific considerations specific to inclusivity in global research is included in the (S1 Checklist).

### Molecular sample processing and bioinformatics

The QIAamp PowerFecal DNA Kit (Qiagen) was used to extract DNA from fecal material, following the manufacturer’s instructions. Before extraction, ethanol was removed from the samples by decanting following 30 min of centrifugation at 12,000 rpm and heated at 50 °C until the ethanol was vaporized. DNA extraction was performed by the Genomics and NGS Core Facility at the Centro de Biología Molecular Severo Ochoa (CBMSO, CSIC-UAM, Spain). To amplify prey DNA, we used two marker sets: a universal eukaryote 18S marker (miniB18S_81 [45]), and the arthropod marker ZBJ [46] for COI region. We selected the 18S marker because steppe birds can feed on a wide range of invertebrates (insects, spiders, snails), and they may also feed on plants (although this seems to be relevant mainly during the non-breeding season, e.g., [33]), so we were initially interested in amplifying both the invertebrate and the plant components of the diet. On the other hand, we selected ZBJ due to its capability to detect a wide range of arthropod prey and to make high taxonomic resolution identifications in metabarcoding studies using fecal samples [46–48]. The combination of both markers in a multi-marker approach was intended to overcome the potential problems associated with marker bias and taxonomic resolution. Each marker was amplified in an independent PCR reaction, in which a negative PCR control (DNA-free) was included. All primers were modified to contain Illumina adaptors at the 5′ end of the sequence (forward primers: 5′‐ACACTGACGACATGGTTCTACA‐3′, reverse primers: 5′‐TACGGTAGCAGAGACTTGGTCT‐3′). For both markers, 30 pg of DNA were used as input in a first PCR of 35 cycles, with a High-Fidelity DNA Polymerase in the presence of 200 nM primers. After the first PCR, a second PCR of 12 cycles was performed with a High-Fidelity DNA Polymerase in the presence of primers (5’-AATGATACGGCGACCACCGAGATCT-[10 nucleotides barcode]-ACACTGACGACATGGTTCTACA-3’ and 5’-CAAGCAGAAGACGGCATACGAGAT-[10 nucleotides barcode]-TACGGTAGCAGAGACTTGGTCT-3’) of the Access Array Barcode Library for Illumina Sequencers (Fluidigm). PCR products were validated and quantified by Bioanalyzer and an equimolar pool was made, purified using AMPure XP beads (Beckman Coulter) and titrated by PicoGreen. Amplicons pools were denatured prior to be seeded on a flow cell where clusters were formed and sequenced using a MiSeq sequencer in a 2x300 pair-end sequencing run (Illumina). Amplification, library preparation and sequencing were carried out by the Genomics Unit of the Fundación Parque Científico de Madrid (Spain).

Bioinformatic processing of sequencing reads was performed using MJOLNIR pipeline (Metabarcoding Joining Obitools and Linkage Networks In R; pipeline steps in S2 Appendix), with a separate analysis for each molecular marker. We first used OBITools [49] to quality filter and align paired-end Illumina sequences, and then we implemented VSEARCH [50] to remove chimaeras. We clustered the sequences into molecular operational taxonomic units (MOTUs) using swarm [51], which is based on an iterative aggregation of sequences that differ less than a given distance. For the taxonomic assignment, we first created a reference database in ecoPCR format for each molecular marker, obtained from the download of all 18S and COI sequences from GenBank database (NCBI). We then used ecotag from OBITools to match the MOTUs generated to the reference sequences with a similarity equal or higher than 0.95. We finally removed pseudogenes using LULU [52]. MOTUs were identified with the most resolved taxonomic assignment possible, and those identified to species or genus were manually confirmed using the BLAST algorithm (NCBI). We removed every taxa not belonging to Animal kingdom, as well as mammals (human), birds and internal parasites (phyla Nematoda and Platyhelminthes). We discarded plant taxa because we were unable to discriminate between environmental contamination, secondary detection or items that were actively consumed by the birds due to the high diversity of plant MOTUs obtained using the 18S marker [37,53]. We also excluded taxa not considered as potential prey items (mites, ticks, springtails [30,37]). We finally removed MOTUs representing less than 1% of the total number of dietary reads [54] to avoid incorporating false positives resulting from tag-jumping events, and samples with less than 100 dietary reads as they were considered to have failed. Negative controls had less than 100 reads and were also removed.

Finally, for each sample, the dietary information derived from the two molecular markers was combined using a python 3.0 script [37]. The script takes into account the differences in taxonomic resolution provided by the different markers, assuming that a dietary component obtained at a lower taxonomic resolution (e.g., order or family) by one of the marker is the same as a component of the same taxonomic group obtained at a higher resolution by the other marker (e.g., genus or species).

### Statistical analysis

For all statistical analyses, we used presence/absence data at order and family levels instead of read count, due to inherent biases present throughout the metabarcoding workflow, including differential DNA extraction success and PCR amplification rates between taxa detected within the diet [55]. All statistical analyses were carried out in R version 4.3.0 [56]. The study region of Southern Spain was excluded from statistical analyses due to the small sample size (Table 1), although its results on dietary composition are provided in the Results section.

To evaluate the most prevalent prey taxa within Dupont’s Lark diet, we calculated the frequency of occurrence (FOO) at family and order levels, defined as the number of fecal samples in which a family or order was detected divided by the total number of samples. We computed FOO by country, and in the case of Spain, by region, phenology and sex. FOO was not calculated for Moroccan study regions due to their small sample sizes.

In order to test whether Dupont’s Lark diet varies over spatial scales (macro and country-level), the species’ phenology and sex groups, we computed multivariate generalized linear models (MGLMs) using the function *manyglm* in the *mvabund* package [57]. This function fitted the presence/absence data of prey taxa at order and family levels to binomial family generalized linear models (with a “cloglog” link function). In total, eight models were fitted, with the predictor variable being country (two levels: Spain/Morocco), Spanish region (five levels), phenology (two levels: breeding season/non-breeding season), and sex (two levels: male/female), and response being the prey multivariate dietary dataset at family and order level (four models for each level of taxonomic classification). To establish appropriate comparisons, in the spatial models we used only male samples, as female samples were not available from all regions. Similarly, in the spatial and sex models we used only the samples collected during the breeding season, since samples from the non-breeding season were only available for males from a single Spanish region (WIR). In the phenological model, therefore, we used only the samples collected in this region in 2017. Furthermore, in order not to introduce confounding effects in the model for testing sex differences, we only used samples from Spanish regions where both male and female samples were available (see Table 1). The significance of variables was determined via likelihood ratio test using the *anova*.*manyglm* function with Monte Carlo resampling (999 iterations) and corrected univariate *p* values for multiple testing. When necessary, we performed pairwise comparisons using the *pairwise*.*comp* function of *anova*.*manyglm*. In addition, *p* values from univariate tests were extracted using the *p*.*uni = “adjusted”* argument to assess if any specific prey taxa were responsible for the spatial, temporal and sexual dietary differences. We visualized dietary differences using non-metric multidimensional scaling analysis (NMDS) based on Jaccard distance of prey families via the function *metaMDS* in the *vegan* package [58].

To quantify overlap in observed Dupont’s Lark diet between countries, Spanish regions, phenological periods and sex groups, we calculated Pianka’s index of dietary overlap [59] using the *EcoSimR* package [60], which allows its calculation from occurrence data. This index quantifies the degree of similarity between two diets and ranges from 0 to 1, where 1 indicates complete overlap. In *EcoSimR*, a null model simulation, based on randomization of dietary data (here based on FOO) is generated and used in a statistical comparison to test whether the observed niche overlap differs from what would be expected by chance. We used the randomization algorithm 3 (RA3), which is a permutation test recommended for niche overlap studies that reshuffles zero and non-zero values [61], and 9999 repetitions for the simulation.

## Results

We successfully amplified invertebrate prey DNA from 199 out of 303 fecal samples (69% in Spain, *n* = 171; 51% in Morocco *n* = 28; Table 1). After all filtering steps, sequences obtained from the ZBJ marker were clustered into 91 dietary MOTUs, with a mean of 9619.8 ± 5850 standard deviation (SD) diet reads and 2.6 ± 1.3 SD MOTU per individual, while sequences obtained from the 18S marker were clustered into 103 dietary MOTUs, with a mean of 11,956 ± 4884 diet reads and 2.6 ± 1.5 SD MOTU per individual. Combined results from both metabarcoding data sets produced 623 occurrences of prey from 185 MOTUs, with a mean of 3.1 ± 1.8 SD MOTU per individual. The obtained MOTUs were identified to 62 families of 12 orders of three classes from the phylum Arthropoda (Insecta, Arachnida and Diplopoda) and one class from the phylum Mollusca (Gastropoda), with 37% of MOTUs identified to species level and 55% to genus (Table I in S3 Appendix). During the breeding season, the most frequent prey order in the diet of Dupont’s Lark (considering both Spanish and Moroccan samples) was Coleoptera (FOO: 60.3%), followed by Lepidoptera (40.7%), Julida (33.2%) and Orthoptera (29.2%). The families Julidae (33.2%), Noctuidae (29.2%), Acrididae (28.6%) and Tenebrionidae (26.1%) dominated Dupont’s Lark diet.

### Variation in diet composition at macro-spatial scale

**Fig 3:**
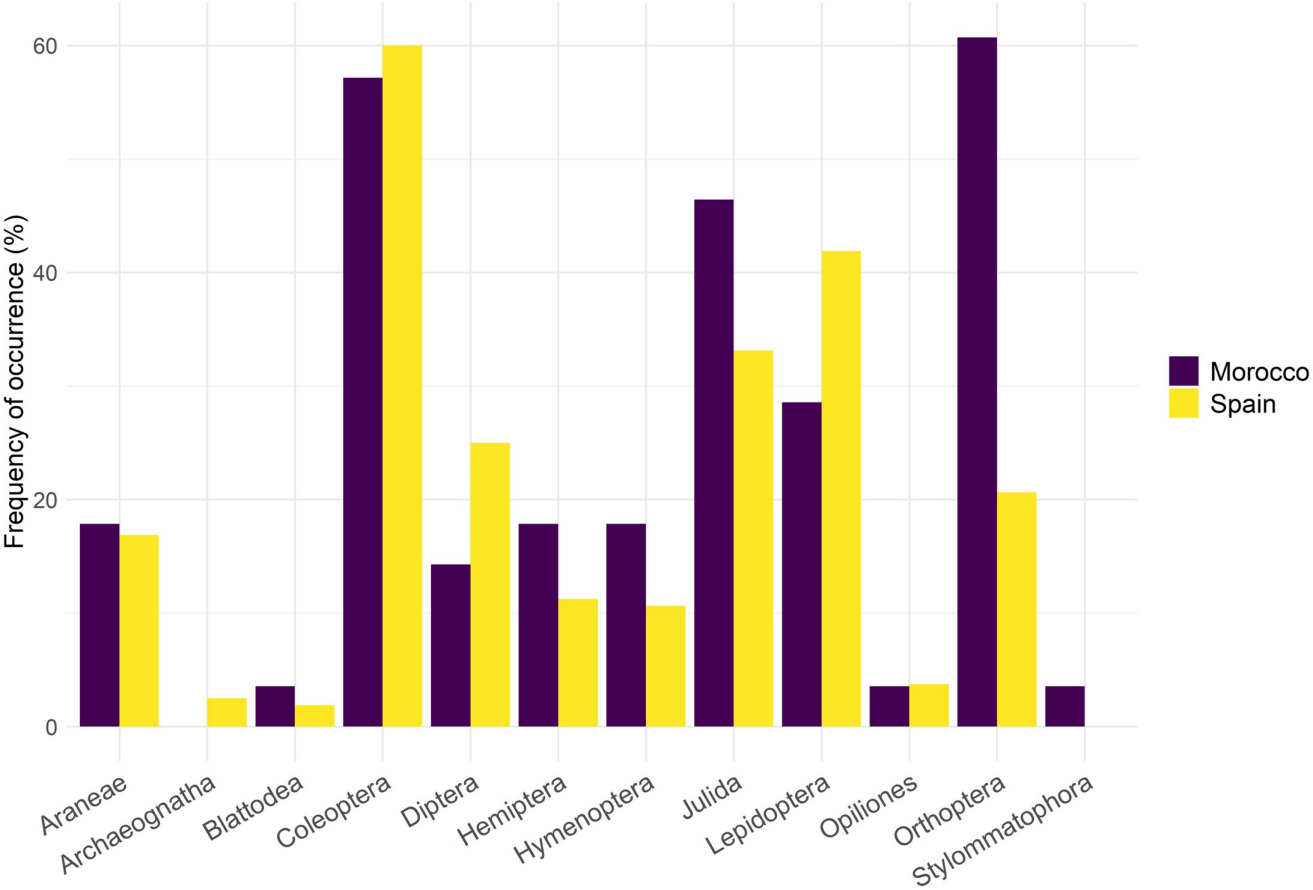
Frequency of occurrence (%) of prey orders in the diet of Dupont’s Lark during the breeding season identified using DNA metabarcoding on Moroccan (*n* = 28) and Spanish (*n* = 160) fecal samples.

**Fig 4:**
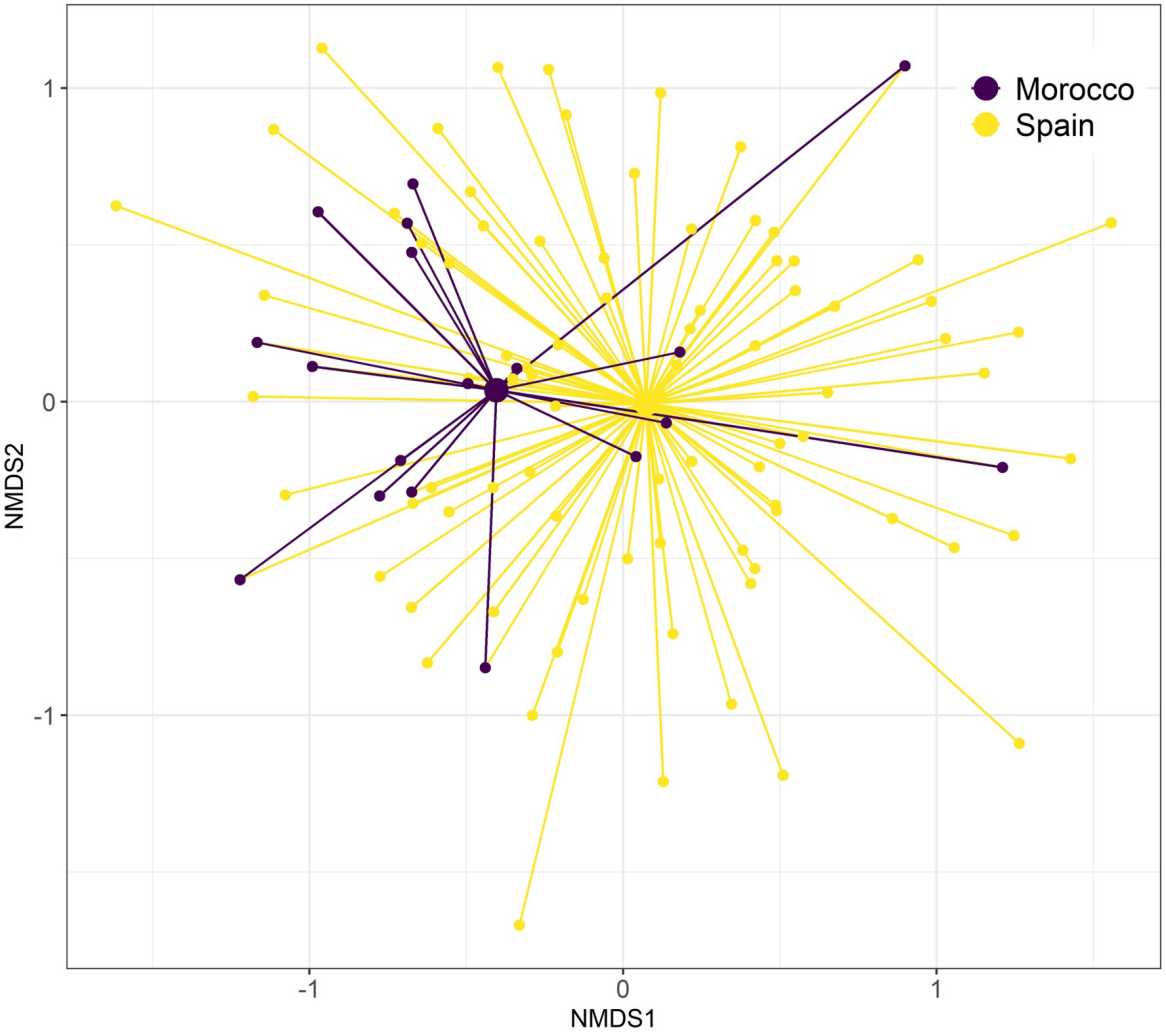
Spider plot of the non-metric multidimensional scaling (NMDS; k = 2, stress = 0.112) for arthropod prey families consumed by Dupont’s Lark in Morocco and Spain. Smaller nodes represent individual birds with connecting lines joining the individual to the mean centroid (larger nodes) of its country.

MGLMs revealed that Dupont’s Lark diet during the breeding season differed significantly between countries (i.e., continents) at both order (LRT Deviance = 30.8, *p* = 0.002) and family levels (LRT Deviance = 97.5, *p* = 0.001). Univariate tests showed that differences at the order level were due to the order Orthoptera (Table I in S4 Appendix), which was detected with higher frequency in Moroccan samples (Fig 3). The families Acrididae and Geometridae were the main drivers of the differences at the family level (Table I in S4 Appendix), with the first family being consumed more frequently by Moroccan Dupont’s larks and the second family by Spanish ones (S1 Table). Despite these differences, niche overlap analysis showed a greater overlap than expected by chance (*p* < 0.001), with a high value of Pianka’s index for arthropod families (0.75), consistent with the diet overlap observed in the NMDS (Fig 4).

### Variation in diet composition at country-level scale

**Fig 5:**
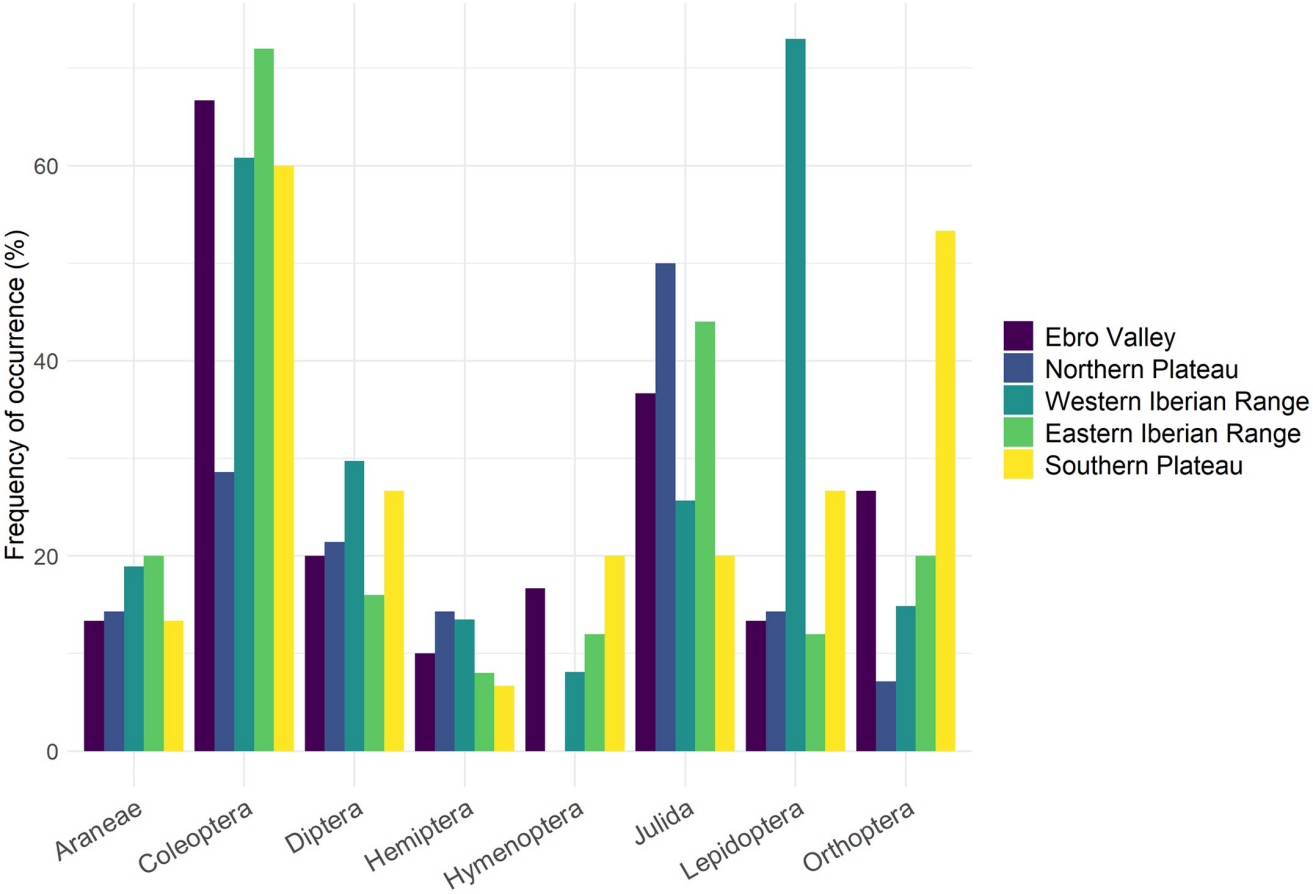
Frequency of occurrence (%) of the eight most common prey orders in the diet of Dupont’s Lark identified using DNA metabarcoding on the fecal samples from the Spanish regions sampled.

MGLMs indicated that Dupont’s Lark diet during the breeding season differed significantly between Spanish regions at both order (LRT Deviance = 111.9, *p* = 0.001) and family levels (LRT Deviance = 321.5, *p* = 0.001). Pairwise comparisons revealed significant differences between the WIR region and all the other regions at the order level (Table II in S4 Appendix). These differences were due to the order Lepidoptera (Table II in S4 Appendix), detected with higher frequency in the samples of the WIR region than in the rest of regions (Fig 5; S1 Table). At the family level, pairwise comparisons showed significant differences between the WIR region and all other regions, and between EV region and NP and SP regions (Table II in S4 Appendix). The lepidopteran families Noctuidae and Geometridae and the coleopteran family Tenebrionidae were significant in the univariate tests (Table II in S4 Appendix), with birds sampled in the WIR region showing the highest frequency for the lepidopteran families (59.5% and 32.4%, respectively; S1 Table), while birds from the EV region for Tenebrionidae. The two Dupont’s Lark individuals with dietary results from the SS region (not included in statistical analysis due to low sample size) consumed only prey items of the families Julidae and Tachinidae (Diptera).

**Fig 6:**
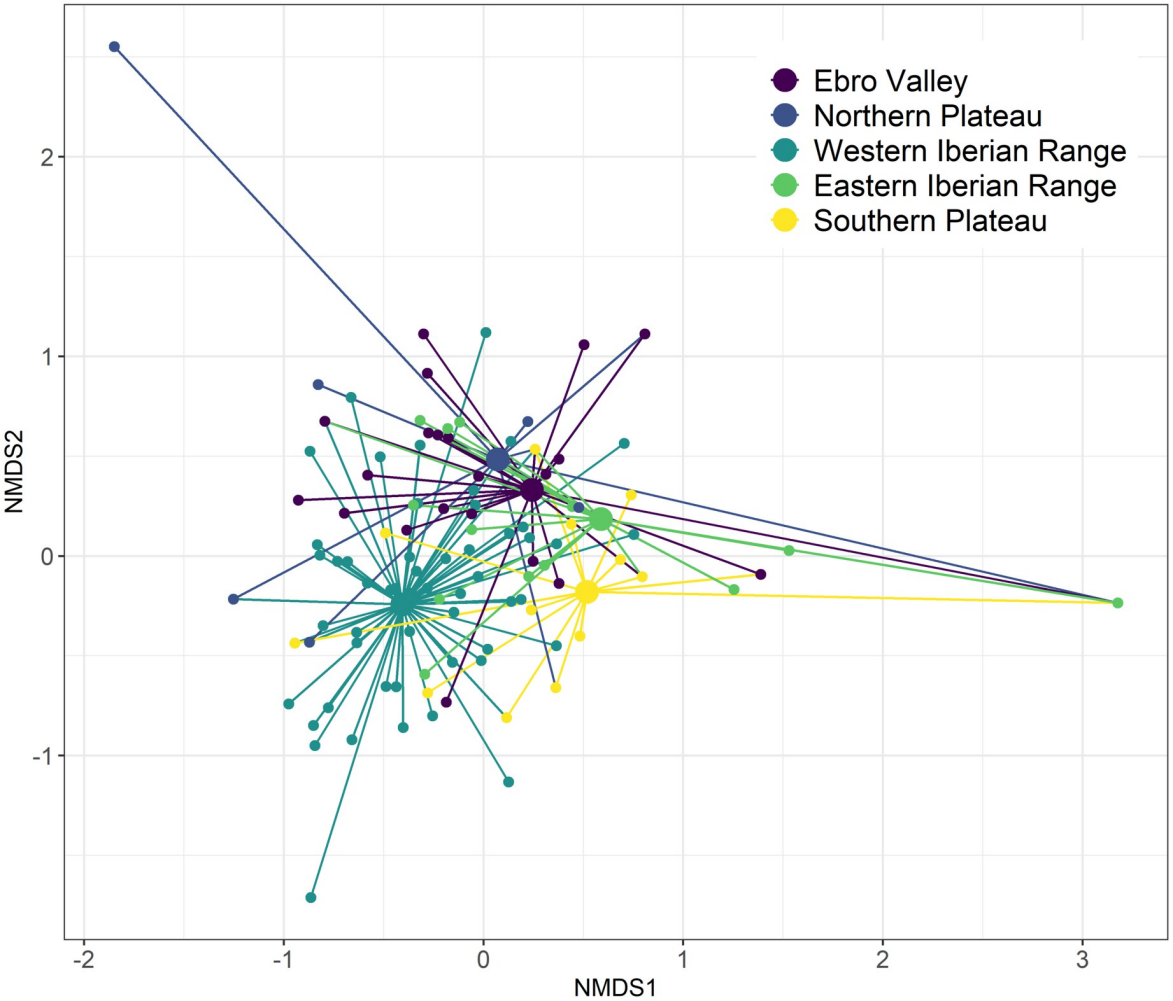
Spider plot of the non-metric multidimensional scaling (NMDS; k = 2, stress = 0.101) for arthropod prey families consumed by Dupont’s Lark across the five Spanish regions. Smaller nodes represent individual birds with connecting lines joining the individual to the mean centroid (larger nodes) of its region.

Niche overlap analysis returned a high degree of dietary overlap among Spanish regions, with a mean value of Pianka’s index of 0.62 and greater than the null expectation (*p* < 0.001), indicating a higher overlap in the consumption of arthropod families than expected by chance. In pairwise comparisons (Table 2) all Pianka’s index values were greater than 0.52 and statistically significant (*p* < 0.05). The lowest dietary niche overlap values were found between the WIR region and all other regions, while the highest values were observed between the EIR region and the EV and NP regions (0.91 and 0.75, respectively). The NMDS (Fig 6) also showed a generally high overlap between most regions, but a higher segregation was observed between the WIR region and the remaining regions and between the EV and SP regions.

**Table 2:** Pairwise Pianka’s index values for niche overlap estimation between Spanish regions based on the frequency of arthropod families consumed by Dupont’s Lark.

	**Ebro Valley**	**Northern Plateau**	**Western Iberian Range**	**Eastern Iberian Range**
Northern Plateau	0.61*			
Western Iberian Range	0.55*	0.52*		
Eastern Iberian Range	0.91***	0.75***	0.52*	
Southern Plateau	0.58*	0.62*	0.54*	0.65**

All values indicate statistically significant niche overlap (i.e. greater than expected by chance based on comparison with 9999 null models;

* p = 0.05 to p = 0.01,

** p = 0.01 to p = 0.001,

*** p < 0.001).

### Variation in diet composition between phenological periods

We found differences in prey composition between the breeding and non-breeding seasons within the WIR region at both order (MGLM: LRT Deviance = 30.4, *p* = 0.001) and family levels (MGLM: LRT Deviance = 54.2, *p* = 0.006). Univariate tests revealed that order Orthoptera and family Acrididae were the main drivers of the differences (Table III in S4 Appendix), with both taxa being detected with higher frequency in the samples collected in the non-breeding season (72.23% vs 10.91% for both taxa; S1 Table). Despite these differences, niche overlap analysis at the family level showed a high overlap, with a Pianka’s index value of 0.60 and greater than expected by chance (*p* < 0.05). The diet overlap was observed in the NMDS (S1 Fig).

### Variation in diet composition between sexes

There were no differences in diet composition between sexes in the breeding season in Spain at either the order (MGLM: LRT Deviance = 6.0, p = 0.89) or family level (MGLM: LRT Deviance = 40.3, p = 0.10). A high Pianka’s index value of 0.85 was obtained in the niche overlap analysis, greater than the null expectation (*p* < 0.001), and consistent with the high diet overlap observed in the NMDS (S2 Fig). Males and females consumed the orders Coleoptera, Lepidoptera and Julida with high frequency (S1 Table). Diptera was detected with higher frequency in female samples (41.7% vs 23.8%; S1 Table), as well as the Coleoptera families Carabidae and Curculionidae (S1 Table).

## Discussion

Here we used a metabarcoding approach to characterize the temporal and spatial variation of the diet of a passerine throughout most of its distribution range. More specifically, our study provides the first comprehensive comparison of dietary composition between Dupont’s Lark populations at macro-spatial (Morocco and Spain) and country-level (within Spain) scales. It also highlighted temporal differences in the diet of Dupont’s Lark across the species’ phenological periods, whereas no dietary differences related to sex were detected. Dietary variation across landscapes and periods probably reflects spatial and temporal differences in the prey community, as well as changes in prey preferences of this bird species that has been shown to exhibit diet selection [35]. This detailed dietary information is essential for understanding species’ ecology and implementing effective conservation interventions [62], especially critical for a species in such a marked population decline [32].

### Dietary composition

Dupont’s Lark can be described as a generalist insectivorous species throughout its distribution range. Larks predated on a wide range of prey groups, but most frequently fed on nutrient-rich arthropods such as beetles, lepidopterans, millipedes, and grasshoppers [63,64]. Other important prey groups were dipterans, spiders, and hemipterans. The few previous dietary studies, based on visual examination of fecal and gizzard contents, also reported the importance of beetles and grasshoppers in the diet of Dupont’s Lark adults [33,65], but did not observe such a high frequency of lepidopteran consumption, and did not report millipede consumption at all. In the case of Lepidoptera, this may be a consequence of the difficulties in visual identification of soft-bodied prey that leave few or no hard parts [37], which highlights the value of using molecular techniques such as metabarcoding for dietary studies of insectivorous predators. In this line, recent metabarcoding studies conducted in Central Spain on the food niche of shrub-steppe birds [14,30] also pointed out the high frequency of consumption of beetles, grasshoppers, millipedes and spiders by Dupont’s Lark, although in the present study, which considers a larger distribution range, lepidopterans were much more often detected in the feces of the species. In addition, we detected here dietary items that had not been described in the previous studies in the diet of adults of this species, such as millipedes of the genus *Cylindroiulus*, frequently consumed in several Spanish regions, or the spider family Oxyopidae, only present in Moroccan samples. It is also remarkable the observed consumption of gastropod prey (snails) both in Spain and Morocco, but especially in the latter country, where the frequency of occurrence reached 10%. Snails represent a good source of nutrients [66] and are an abundant prey in Moroccan steppes [67]. References on snail consumption in larks are very limited (but see [68]).

### Spatial variation

Spatial dietary differences of Dupont’s Lark were observed at different scales, probably reflecting dietary plasticity in response to variations in prey availability [69,70]. At a macro-spatial scale, the diet of Dupont’s Lark varied between Spain and Morocco, with greater frequency of grasshoppers (Acrididae, Orthoptera) in the diet of African birds. This may be related to habitat differences between countries, with Moroccan regions sampled dominated by alfa grass steppes, in contrast to the low scrub vegetation dominant in the Iberian steppes. Since the spatial distribution and abundance of many grasshopper species are closely linked to herbaceous vegetation [71,72], which they use as food and shelter, it may be possible that the presence of grasshopper species is higher in the grass steppes inhabited by Dupont’s Lark in Morocco than in the Iberian shrub-steppes. Also, the phenology of grasshoppers may be an important factor in explaining the dietary differences found, since in Spain, the sampling time (early March-early June) was probably previous to the peak of adult grasshoppers’ activity, which in temperate regions usually starts in summer [73], while in Morocco, warmer conditions due to climate change have been reported to accelerate grasshopper development of species that show their peak in spring [74].

Dietary differences between continents might be also associated to morphological variations between Spanish and Moroccan populations. Feeding traits varied within the Dupont’s lark range probably in response to different natural selection forces acting in each part of the range [75]. Previous studies found that bill length and volume and tarsus length of Moroccan Dupont’s Larks were larger than in Spanish birds [75,76], which likely increases accessibility to a broader range of prey species of different sizes [77]. These dietary differences were probably not sufficient to lead to a country-level diet differentiation, reflected in the high degree of overlap, since at both sites individuals fed with similar frequency on other prey groups (beetles, lepidopterans, millipedes and spiders).

At country-level scale, the diet of Dupont’s Lark varied spatially among regions, probably influenced by landscape characteristics, which strongly shape the availability of insects and other arthropods, especially through features such as plant species richness or landscape heterogeneity [78]. The Spanish region of Western Iberian Range appeared to be the most differentiated, with lower diet overlap values, and primarily due to a notably higher frequency of lepidopteran consumption, especially from the families Noctuidae and Geometridae. These results are in line with the distributions of the main consumed lepidopterans of these families, such as species of the noctuid genus *Agrotis* (e.g., *Agrotis pierreti*), which present their principal Iberian populations in the Western Iberian Range [79,80]. Other Spanish regions also showed dietary variation, specifically the Ebro Valley and the plateau regions, mainly due to the higher frequency of consumption of Tenebrionidae beetles by Dupont’s larks from the Ebro Valley. This might be related to the high adaptability of Tenebrionidae beetles to xeric conditions, being a dominant taxa in arid and semi-arid systems [81] such as the Ebro Valley region, which is one of the most arid areas in Spain and Europe [82]. However, monitoring arthropod availability in occupied areas should be essential to better understand the dietary differences found, as well as to investigate prey selection throughout Dupont’s Lark distribution (see e.g., [35]). It is important to note that these results are derived from an unbalanced sample size among regions. Specifically, the Western Iberian Range region had notably larger sample size than the other regions. This discrepancy may have impacted our findings, as samples from those areas may have included taxa absent in samples from other sites due solely to the sample size effect. Consequently, interpretations should be treated with caution.

### Phenological variation

As hypothesized, we found differences in the diet composition of Dupont’s Lark between breeding and non-breeding seasons in Spain, primarily due to an increase in the frequency of consumption of grasshoppers of the family Acrididae during the non-breeding period. This variation may reflect changes in grasshopper availability and richness [8], as most acridid species in the Iberian Peninsula start their adult activity period at early summer, many of them reaching their maximum population sizes in September and October [83]. Although not significant, we also found temporal differences in the millipede consumption, as this arthropod group was not present in the non-breeding samples, period in which Mediterranean species reproduce and burrow until the adults emerge in spring [84]. Despite the differences, a high degree of dietary overlap was observed between the species’ phenological periods, since important arthropod groups in the diet of the species were predated during both periods with similar frequencies (e.g., Carabidae, Curculionidae, Tenebrionidae, Geometridae or Lycosidae), which might be related to the resident status of the species [85]. Future work should provide information on changes in prey availability in the study area between periods to better understand temporal variations in the diet of the species.

Temporal dietary changes have also been reported for other insectivorous bird species [8,48], but to our knowledge, this study is the first to describe this pattern for a steppe passerine species. However, these results should be taken with caution due to the small sample size of the non-breeding period, so future work should incorporate a greater number of samples and extend the temporal coverage to the annual period. The only previous work on the non-breeding diet of Dupont’s Lark, based on the observation of four gizzard contents of specimens from the Ebro Valley, indicated the consumption of seeds of three plant genera in addition to insects [33]. The consumption of plant material in autumn and winter appears to be a common strategy in other steppe larks such as the Eurasian Skylark (*Alauda arvensis* [86,87]. In this study, however, the plant component of the diet of Dupont’s Lark has not been analyzed, since the high diversity of plant MOTUs obtained with the marker 18S made it difficult to discriminate between environmental contamination, secondary consumption or items that were actively consumed by the birds [37,53]. Nonetheless, detailed information on plant consumption might also be essential for a complete understanding of the dietary ecology of the species, and especially relevant for designing conservation strategies during the non-breeding season, when the plant fraction of the diet may acquire greater importance.

### Sexual variation

Sexual dietary differences have already been described for other bird species, including generalist medium-sized passerines such as the Black Wheatear (*Oenanthe leucura* [16] or the Hawfinch (*Coccothraustes coccothraustes* [70]). In contrast to our predictions, we revealed a high overlap between sexes in the diet of Dupont’s Lark during the breeding season, suggesting that foraging behavior and nutritional requirements are probably equivalents in both sexes. During the breeding season, females and males are expected to have different energetic demands due to differential reproductive roles [17], with Dupont’s Lark females implicated in nest-building, egg-laying and incubating, although both sexes are involved in food provisioning to nestlings [88]. This may result in females facing a stronger trade-off between self-maintenance and breeding [89]. A plausible explanation might be that abundance of resources in the immediate nest surroundings is higher than elsewhere, enabling females to prey on arthropod groups with similar frequencies to males’, although direct comparisons of arthropod availability around and far from the nest would be required. Therefore, the absence of sexual diet differentiation suggests that food availability may not be a limiting factor during the breeding season in the Iberian steppes [90]. However, although both sexes preyed on the same arthropod groups, it may be possible that males and females fed on prey of different size [91], which cannot be evaluated with the method used in this study.

It is important to note that these conclusions are based on a small number of samples from females (*n* = 12), since the trapping method was inherently biased towards males [43]. Future research should aim to increase the sample size of females using more effective methods for their capture (e.g., passive-trap around the nest or thermal cameras), as well as consider complementary methods able to determine the size of prey consumed by each sex, in order to draw more robust inferences about sexual variation in Dupont’s Lark diet. Nevertheless, our findings provide a first approximation into the diet of female Dupont’s larks and about the dietary patterns of the species, essential to integrate the female fraction of the population in conservation strategies.

### Metabarcoding considerations

In any metabarcoding study, the selection of the most appropriate primer set is critical and can affect the results obtained and the subsequent ecological interpretations [92]. Here, we used a multi-marker approach combining a universal marker (18S) with a specific arthropod marker (ZBJ), which allowed us to minimize the problems of taxonomic resolution and the potential taxonomic biases of the different markers [93]. The 18S amplified a broader taxonomic diversity than the ZBJ, but at the expense of higher taxonomic resolution, whereas the ZBJ probably biased detection in favor of Lepidoptera and Diptera over other arthropod taxa [37], failing to detect three entire prey orders that 18S detected (Blattodea, Archaeognatha and Stylommatophora). Although ZBJ usually provides identifications with high taxonomic resolution, we only identified 37% of the MOTUs at the species level, so dietary comparisons were performed at the order and family level. Therefore, dietary overlap and differences in prey composition for all the factors studied were conditioned by the level of identification reached. Had we been able to perform dietary comparisons at the species level, we would probably have detected greater differences and lower overlaps in our results.

Furthermore, metabarcoding is unable to discriminate life stages of consumed arthropods (adults, larvae or eggs), essential information for a comprehensive knowledge of the food ecology of Dupont’s Lark. Finally, our dietary study was based on presence/absence data rather than on read counts, since DNA-based methods remain subjected to a variety of factors (e.g. differential digestion rates of tissues, differences of copy numbers of marker genes between tissue types or prey taxa, PCR primer bias) that prevent to accurately correspond reads to the amount of each prey item consumed [38]. These and other considerations, including sample preservation and storage conditions, the incorporation of biological and technical replicates, or the use of negative and positive controls, should be taken into account when designing a DNA metabarcoding study to provide representative results of species’ diet.

### Conclusions and implications for Dupont’s Lark conservation

Metabarcoding represents a methodological advance for the study of insectivorous bird diets, providing greater accuracy for the identities and frequencies of prey taxa consumed compared to traditional techniques [37]. This study provides the first molecular insight into the diet of Dupont’s Lark across its European and Moroccan distribution range. The species fed on a wide range of arthropod prey, showing dietary differences between countries, regions and phenological periods, probably reflecting the plasticity and adaptability of the species to temporal and landscape differences in resource availability. Dietary plasticity can be important to this critically endangered bird [32], as it means that the species has certain ability to cope with unusual challenges such as food shortages [94]. However, arthropods are experiencing global declines in diversity and abundance in recent decades [95,96], mostly due to habitat loss and degradation, and to agricultural intensification (pesticide usage, increased use of fertilizers and frequency of agronomic measures [97,98]). The loss of arthropod biomass is expected to provoke cascading effects on food webs [95], raising concern about food limitations that could adversely affect the viability of insectivorous birds [99]. Furthermore, climate change is known to have the potential to alter synchrony between food availability and timing of reproduction in birds [100], which may have also important consequences for Dupont’s Lark populations. For instance, warmer spring temperatures in Europe have been shown to cause seasonal shifts to earlier activity in several key groups for Dupont’s Lark (e.g., Noctuidae [101] or Carabidae [102]), potentially leading to problems of mismatch with the breeding phenology of this bird species. We also found that the diet of this species does not vary between sexes during the breeding season, suggesting similar food requirements and foraging habits of males and females, although additional research at a broader spatial scale and with a larger female sample size would be needed to further investigate sexual dietary patterns. Finally, the results of this study provide valuable information for the conservation of Dupont’s Lark and other insectivores of the steppe habitat. Despite being described as a generalist insectivore, the promotion of specific prey taxa shown in this study to be relevant in specific areas of the range (e.g., Acrididae or Noctuidae in Morocco and the Western Iberian Range, respectively) and of periods of the year (Acrididae during the non-breeding season) might be essential to the conservation of this bird species across its distribution area and throughout the year. In this regard, conservation and management measures focused on maintaining the quality of steppe habitat, including the promotion of traditional practices such as extensive sheep grazing, are recommended. Extensive sheep grazing at intermediate levels of intensity favors the availability of a variety of arthropod groups, especially coprophagous and epigeous [29], which include key prey in the diet of Dupont’s Lark throughout its range (e.g., species of the orders Coleoptera or Orthoptera). An action to promote extensive sheep grazing in Spanish steppes is the establishment of grazing regimes through voluntary agreements with local farmers under land stewardship programs [103], whereas in the Moroccan steppes, the intensification of livestock systems supported by agricultural policies and the decrease in forage availability due to recurrent drought poses additional challenges [104].

## Supporting information

S1 Checklist Inclusivity in global research questionnaire. (DOCX)

S1 Table Frequency of occurrence (%) of prey orders and families in the diet of Dupont’s Lark calculated for each country, sex, phenological period and Spanish region. Values are color coded according to FOO (darker shading indicates higher FOO).
(XLSX)

S1 Fig Spider plot of the non-metric multidimensional scaling (NMDS; k = 2, stress = 0.139) for arthropod prey families consumed by Dupont’s Lark in the breeding and non-breeding seasons. Smaller nodes represent individual birds with connecting lines joining the individual to the mean centroid (larger nodes) of its phenological period.
(TIF)

S2 Fig Spider plot of the non-metric multidimensional scaling (NMDS; k = 2, stress = 0.109) for arthropod prey families consumed by Dupont’s Lark males and females in the breeding season. Smaller nodes represent individual birds with connecting lines joining the individual to the mean centroid (larger nodes) of its sex.
(TIF)

S1 Appendix Location of Moroccan sampling points and sample size. (DOCX)

S2 Appendix Bioinformatic pipeline description. (DOCX)

S3 Appendix Prey items detected with the multi-marker approach and sample metadata. (XLSX)

S4 Appendix MGLMs for differences in prey composition. (XLSX)

## References

[1] Robbins CT. Wildlife feeding and nutrition, 4th edn. Academic Press, New York; 1993.

[2] Durst SL, Theimer TC, Paxton EH, Sogge MK. Age, habitat, and yearly variation in the diet of a generalist insectivore, the Southwestern Willow Flycatcher. Condor. 2008;110(3): 514–525.

[3] Begg CM, Begg KS, Du Toit JT, Mills MGL. Sexual and seasonal variation in the diet and foraging behaviour of a sexually dimorphic carnivore, the honey badger (*Mellivora capensis*). J Zool. 2003;260(3): 301–316.

[4] Clare EL, Barber BR, Sweeney BW, Hebert PDN, Fenton MB. Eating local: influences of habitat on the diet of little brown bats (*Myotis lucifugus*). Mol Ecol 2011;20(8): 1772–1780. 21366747 10.1111/j.1365-294X.2011.05040.x

[5] Murray SW, Kurta A. Spatial and temporal variation in diet. In: Kurta A, Kennedy J, editors. The Indiana bat: Biology and management of an endangered species. Austin, TX: Bat Conservation International; 2002. pp. 182–192.

[6] Pollard KA, Holland JM. Arthropods within the woody element of hedgerows and their distribution pattern. Agric For Entomol. 2006;8(3): 203–211.

[7] Beck ML, Hopkins WA, Jackson BP. Spatial and temporal variation in the diet of Tree Swallows: implications for trace-element exposure after habitat remediation. Arch Environ Contam Toxicol. 2013;65(3): 575–587. doi: 10.1007/s00244-013-9913-5 23695717

[8] Davies SR, Vaughan IP, Thomas RJ, Drake LE, Marchbank A, Symondson WOC. Seasonal and ontological variation in diet and age-related differences in prey choice, by an insectivorous songbird. Ecol Evol. 2022;12: e9180. doi: 10.1002/ece3.9180 35979519 PMC9366593

[9] Tournayre O, Leuchtmann M, Galan M, Trillat M, Piry S, Pinaud D, et al. eDNA metabarcoding reveals a core and secondary diets of the greater horseshoe bat with strong spatio-temporal plasticity. Environ DNA. 2021;3(1): 277–296.

[10] Pyke GH, Pulliam HR, Charnov EL. Optimal foraging: a selective review of theory and tests. Q Rev Biol. 1977;52: 137–154.

[11] Ramos R, Ramírez F, Carrasco JL, Jover L. Insights into the spatiotemporal component of feeding ecology: an isotopic approach for conservation management sciences. Divers Distrib. 2011;17(2): 338–349.

[12] Wellicome TI, Danielle Todd L, Poulin RG, Holroyd GL, Fisher RJ. Comparing food limitation among three stages of nesting: supplementation experiments with the burrowing owl. Ecol Evol. 2013;3(8): 2684–2695. doi: 10.1002/ece3.616 24567832 PMC3930041

[13] Navarro J, Kaliontzopoulou A, González-Solís J. Sexual dimorphism in bill morphology and feeding ecology in Cory’s shearwater (*Calonectris diomedea*). Zool. 2009;112(2): 128–138. 10.1016/j.zool.2008.05.001 18950998

[14] Zurdo J, Gómez-López P, Barrero A, Bustillo-de la Rosa D, Gómez-Catasús J, Reverter M, et al. Selecting the best: Interspecific and age-related diet differences among sympatric steppe passerines. Avian Res. 2023;14: 100151.

[15] Bravo C, Ponce C, Bautista LM, Alonso JC. Dietary divergence in the most sexually size-dimorphic bird. Auk. 2016;133(2): 178–197.

[16] da Silva LP, Mata VA, Lopes PB, Lopes RJ, Beja P. High-resolution multi-marker DNA metabarcoding reveals sexual dietary differentiation in a bird with minor dimorphism. Ecol Evol. 2020;10(19): 10364–10373. doi: 10.1002/ece3.6687 33072265 PMC7548197

[17] Catry P, Phillips RA, Croxall JP. Sexual segregation in birds: patterns, processes and implications for conservation. In: Ruckstuhl KE, Neuhaus P, editors. Sexual segregation in vertebrates: ecology of the two sexes. Cambridge, UK: Cambridge University Press; 2005. pp. 351–378.

[18] Bolnick DI, Doebeli M. Sexual dimorphism and adaptive speciation: two sides of the same ecological coin. Evol. 2003;57: 2433–2449. doi: 10.1111/j.0014-3820.2003.tb01489.x 14686521

[19] Wiens JA. Fat times, lean times and competition among predators. TREE 1993;8: 348–349. doi: 10.1016/0169-5347(93)90216-C 21236192

[20] García JT, Suárez F, Garza V, Justribó JH, Oñate JJ, Hervás I, et al. Assessing the distribution, habitat, and population size of the threatened Dupont’s Lark *Chersophilus duponti* in Morocco: Lessons for conservation. Oryx. 2008;42: 592.

[21] BirdLife International. Species factsheet: *Chersophilus duponti*; 2023. http://datazone.birdlife.org/species/factsheet/duponts-lark-chersophilus-duponti on 07/10/2023.

[22] Traba J, Pérez-Granados C, Serrano D. Alondra ricotí. *Cherosphilus duponti*. In: López-Jiménez J, editor. Libro Rojo de las Aves de España. SEO/BirdLife, Madrid; 2021. pp. 321–329.

[23] Garza V, Suárez F, Herranz J, Traba J, García de la Morena EL, Morales MB, et al. Home range, territoriality and habitat selection by the Dupont’s lark *Chersophilus duponti* during the breeding and postbreeding periods. Ardeola. 2005;52: 133–146.

[24] Laiolo P, Tella JL. Fate of unproductive and unattractive habitats: Recent changes in Iberian steppes and their effects on endangered avifauna. Environ Conserv. 2006;33(3): 223–232.

[25] Traba J, Morales MB. The decline of farmland birds in Spain is strongly associated to the loss of fallowland. Sci Rep. 2019;9: 9473. doi: 10.1038/s41598-019-45854-0 31263119 PMC6603185

[26] Viñuela J, García JT, Suárez F. Marked range regression and possible alteration of distribution of the Dupont’s Lark *Chersophilus duponti* in Tunisia: Conservation consequences of vanishing alfa grass *Stipa tenacissima* steppes in North Africa. Diversity. 2023;15: 549.

[27] Oñate JJ, Suárez F, Calero-Riestra M, Justribó JH, Hervás I, de la Morena ELG, et al. Responses of bird communities to habitat structure along an aridity gradient in the steppes north of the Sahara. Diversity. 2023;15: 737.

[28] García-Antón A, Garza V, Traba J. Connectivity in Spanish metapopulation of Dupont’s lark may be maintained by dispersal over medium-distance range and stepping stones. PeerJ. 2021;9: e11925. doi: 10.7717/peerj.11925 34466286 PMC8380426

[29] Gómez-Catasús J, Reverter M, Bustillo-de la Rosa D, Barrero A, Pérez-Granados C, Zurdo J, et al. Moderate sheep grazing increases arthropod biomass and habitat use by steppe birds. Agric Ecosyst Environ. 2023;354: 108556.

[30] Zurdo J, Barrero A, da Silva LP, Bustillo-de la rosa D, Gómez-Catasús J, Morales MB, et al. Dietary niche overlap and resource partitioning among six steppe passerines of Central Spain using DNA metabarcoding. Ibis. 2023;165(3): 905–923.

[31] Cramp S. Handbook of the Birds of Europe, Middle East and North Africa: The Birds of the Western Palearctic. Volume V. Tyrant Flycatchers to Thrushes: 1063. Oxford: Oxford University Press; 1988.

[32] Reverter M, Pérez-Granados C, López-Iborra GM, García-Mellado A, Aledo-Olivares E, Alcántara M, et al. Range contraction and population decline of the European Dupont’s Lark population. Diversity. 2023;15: 928.

[33] Aragüés A. La alondra de Dupont (*Chersophilus duponti*) en Monegros. Boletín de la S.E.A. 1999;24: 196–198.

[34] Herranz J, Yanes M, Suárez F. Primeros datos sobre la dieta de pollos de Alondra de Dupont, *Chersophilus duponti*, en la Península Ibérica. Ardeola. 1993;40: 77–79.

[35] Zurdo J, Reverter M, Barrero A, Bustillo-de la Rosa D, Gómez-Catasús J, Pérez-Granados C, et al. Prey choice in insectivorous steppe passerines: New insights from DNA metabarcoding. Glob Ecol Conserv. 2023;48: e02738.

[36] Pompanon F, Deagle BE, Symondson WOC, Brown DS, Jarman SN, Taberlet P. Who is eating what: Diet assessment using next generation sequencing. Mol Ecol 2012;21: 1931–1950. doi: 10.1111/j.1365-294X.2011.05403.x 22171763

[37] da Silva LP, Mata VA, Lopes PB, Pereira P, Jarman SN, Lopes RJ, et al. Advancing the integration of multimarker metabarcoding data in dietary analysis of trophic generalists. Mol Ecol Resour. 2019;19: 1420–1432. doi: 10.1111/1755-0998.13060 31332947 PMC6899665

[38] Hoenig BD, Snider AM, Forsman AM, Hobson KA, Latta SC, Miller ET, et al. Current methods and future directions in avian diet analysis. Ornithology. 2022;139(1): ukab077.

[39] Traba J, Gómez-Catasús J, Barrero A, Bustillo-de la Rosa D, Zurdo J, Hervás I, et al. Comparative assessment of satellite- and drone-based vegetation indices to predict arthropod biomass in shrub-steppes. Ecological Applications. 2022;32(8): e2707. doi: 10.1002/eap.2707 35808937 PMC10078389

[40] Suárez F. La alondra ricotí (Chersophilus duponti). Dirección General para la Biodiversidad. Ministerio de Medio Ambiente y Medio Rural y Marino Medio Rural y Marino, Madrid; 2010.

[41] García-Antón A, Garza V, Hernández Justribó J, Traba J. Factors affecting Dupont’s Lark distribution and range regression in Spain. PLOS ONE. 2019;14: e0211549. 30768612 10.1371/journal.pone.0211549 PMC6377091

[42] Bustillo-de La Rosa D, Traba J, Calero-Riestra M, Morales MB, Barrero A, Viñuela J, et al. Recent changes in genetic diversity, structure, and gene flow in a passerine experiencing a rapid population decline, the Dupont’s Lark (*Chersophilus duponti*). Diversity. 2022;14(12): 1120.

[43] Barrero A, Gómez‐Catasús J, Pérez‐Granados C, Bustillo‐de la Rosa D, Traba J. Conspecific density and habitat quality drive the defence and vocal behaviour of a territorial passerine. Ibis. 2023.

[44] Vögeli M, Serrano D, Tella JL, Méndez M, Godoy JA. Sex determination of Dupont’s lark *Chersophilus duponti* using molecular sexing and discriminant functions. Ardeola. 2007;54(1): 69–79.

[45] Cabodevilla X, Gómez-Moliner BJ, Abad N, Madeira MJ. Simultaneous analysis of the intestinal parasites and diet through eDNA metabarcoding. Integr Zool. 2023;18: 399–413. doi: 10.1111/1749-4877.12634 35179820

[46] Zeale MRK, Butlin RK, Barker GLA, Lees DC, Jones G. Taxon-specific PCR for DNA barcoding arthropod prey in bat faeces. Mol Ecol Resour. 2011;11: 236–244. doi: 10.1111/j.1755-0998.2010.02920.x 21429129

[47] Trevelline BK, Nuttle T, Hoenig BD, Brouwer NL, Porter BA, Latta SC. DNA metabarcoding of nestling feces reveals provisioning of aquatic prey and resource partitioning among Neotropical migratory songbirds in a riparian habitat. Oecologia. 2018;187(1): 8598. 10.1007/s00442-018-4136-0 29616401

[48] McClenaghan B, Nol E, Kerr KCR. DNA metabarcoding reveals the broad and flexible diet of a declining aerial insectivore. Auk 2019;136: 1–11.

[49] Boyer F, Mercier C, Bonin A, Le Bras Y, Taberlet P, Coissac E. OBITools: A unix-inspired software package for DNA metabarcoding. Mol Ecol Resour. 2016;16: 176–182. doi: 10.1111/1755-0998.12428 25959493

[50] Rognes T, Flouri T, Nichols B, Quince C, Mahé F. VSEARCH: A versatile open source tool for metagenomics. PeerJ. 2016;4: 1–22. doi: 10.7717/peerj.2584 27781170 PMC5075697

[51] Mahé F, Rognes T, Quince C, de Vargas C, Dunthorn M. Swarm v2: Highly-scalable and high-resolution amplicon clustering. PeerJ. 2015;3: 1–12. doi: 10.7717/peerj.1420 26713226 PMC4690345

[52] Frøslev TG, Kjøller R, Bruun HH, Ejrnæs R, Brunbjerg A.K, Pietroni C, et al. Algorithm for post-clustering curation of DNA amplicon data yields reliable biodiversity estimates. Nat Commun. 2017;8: 1188. doi: 10.1038/s41467-017-01312-x 29084957 PMC5662604

[53] Tercel MP, Symondson WOC, Cuff JP. The problem of omnivory: A synthesis on omnivory and DNA metabarcoding. Mol Ecol. 2021;30: 2199–2206. doi: 10.1111/mec.15903 33772967

[54] Drake LE, Cuff JP, Young RE, Marchbank A, Chadwick EA, Symondson WOC. An assessment of minimum sequence copy thresholds for identifying and reducing the prevalence of artefacts in dietary metabarcoding data. Methods Ecol Evol. 2021;13: 1–17.

[55] Lamb PD, Hunter E, Pinnegar JK, Creer S, Davies RG, Taylor MI. How quantitative is metabarcoding: A meta-analytical approach. Mol. Ecol. 2019;28(2): 420–430. doi: 10.1111/mec.14920 30408260 PMC7379500

[56] R Core Team. R: A Language and Environment for Statistical Computing. R Foundation for Statistical Computing, Vienna, Austria; 2023. https://www.R-project.org/.

[57] Wang Y, Naumann U, Eddelbuettel D, Wilshire J, Warton D. mvabund: Statistical Methods for Analysing Multivariate Abundance Data. R package version 4.2.1; 2022. https://CRAN.R-project.org/package=mvabund.

[58] Oksanen J, Simpson G, Blanchet F, Kindt R, Legendre P, Minchin P, et al. vegan: Community Ecology Package. R package version 2.6–4; 2022. https://CRAN.R-project.org/package=vegan.

[59] Pianka ER. The structure of lizard communities. Annu Rev Ecol Evol Syst. 1973;4: 53–74.

[60] Gotelli NJ, Hart EM, Ellison AM. EcoSimR: Null model analysis for ecological data. R package version 0.1.0; 2015. http://github.com/gotellilab/EcoSimR

[61] Lawlor LR. Structure and stability in natural and randomly constructed model ecosystems. Am Nat. 1980;116: 394–408.

[62] Bedrosian G, Watson JW, Steenhof K, Kochert MN, Preston CR, Woodbridge B, et al. Spatial and temporal patterns in Golden Eagle diets in the Western United States, with implications for conservation planning. J Raptor Res. 2017;51(3): 347–367.

[63] Bureš S, Weidinger K. Sources and timing of calcium intake during reproduction in flycatchers. Oecologia. 2003;137: 634–647. doi: 10.1007/s00442-003-1380-7 14505029

[64] Razeng E, Watson DM. Nutritional composition of the preferred prey of insectivorous birds: popularity reflects quality. J Avian Biol. 2015;46: 89–96.

[65] Talabante C, Aparicio A, Aguirre JL, Peinado M. Avances en el estudio de la alimentación de adultos de Alondra ricotí (*Chersophilus duponti*) y la importancia de los escarabajos coprófagos. I Workshop nacional de la Alondra Ricotí: Estrategias futuras. Estación Ornitológica de Padul (EOP), Granada; 2015.

[66] Diarra SS. Utilisation of snail meal as a protein supplement in poultry diets. Poult Sci J. 2015;71(3): 547–554.

[67] Guennoun FZ, Mostakim L, Ghamizi M. Biodiversity assessment of terrestrial snails (Mollusca, Gastropoda) of Essaouira’ dunes of Morocco: testing factors affecting the distribution of terrestrial molluscs. Appl Ecol Environ Res. 2023;21(3).

[68] Yanes M, Suárez F, Manrique J. La cogujada montesina, *Galerida theklae*, como depredador del caracol *Otala lactea*: Comportamiento alimenticio y selección de presa. Ardeola. 1991;38(2): 297–303.

[69] Shin D-M, Yoo J-C, Jeong D-M. Spatial variation of Eurasian Eagle-Owl diets in wetland and non-wetland habitats in West-Central Korea. J Raptor Res. 2013;47(4): 400–409.

[70] Stenhouse EH, Bellamy P, Kirby W, Vaughan IP, Drake LE, Marchbank A, et al. Multi-marker DNA metabarcoding reveals spatial and sexual variation in the diet of a scarce woodland bird. Ecol Evol. 2023;13: e10089. doi: 10.1002/ece3.10089 37206688 PMC10191781

[71] Badenhausser I, Gross N, Cordeau S, Bruneteau L, Vandier M. Enhancing grasshopper (Orthoptera: Acrididae) communities in sown margin strips: the role of plant diversity and identity. Arthropod-Plant Interact. 2015;9(4): 333–346.

[72] Wei S, Liu X, McNeill MR, Wang Y, Sun W, Tu X, et al. Identification of spatial distribution and drivers for grasshopper populations based on geographic detectors. Ecol Indic. 2023;154: 110500.

[73] Aguirre-Segura A, Barranco P. Orden Orthoptera. Ibero Diversidad Entomológica. 2015;46: 1–13.

[74] Imene BS, Abboud H, Daniel P. Phenology of early-season and mid-season grasshoppers shows contrasted responses toward climatic variations in an arid area. Intern J Zool. 2019;5(1): 22–32.

[75] García JT, Suárez F, Garza V, Calero-Riestra M, Hernández J, & Pérez-Tris J. Genetic and phenotypic variation among geographically isolated populations of the globally threatened Dupont’s lark *Chersophilus duponti*. Mol Phylogenetics Evol. 2008;46(1): 237–251. 10.1016/j.ympev.2007.06.022 17719801

[76] García-Antón A, Garza V, Traba J. Climate, isolation and intraspecific competition affect morphological traits in an endangered steppe bird, the Dupont’s Lark *Chersophilus duponti*. Bird Study. 2018;65(3): 373–384.

[77] Leisler B, Winkler H. Evolution of island warblers: beyond bills and masses. J Avian Biol. 2015:46(3): 236–244.

[78] Bonari G, Fajmon K, Malenovský I, Zelený D, Holuša J, Jongepierová I, et al. Management of semi-natural grasslands benefiting both plant and insect diversity: The importance of heterogeneity and tradition. Agric Ecosyst Environ. 2017;246: 243–252.

[79] Calle JA. Noctuidos españoles. Ministerio de Agricultura, Pesca y Alimentación. Madrid; 1982.

[80] Magro R, Jambrina J. Catálogo razonado de los Lepidoptera de Castilla y León, España (Parte III) (Lepidoptera: Notodontidae, Euteliidae, Noctuidae). SHILAP Rev Lepidopt. 2014;42: 173–212.

[81] Piñero FS, Tinaut A, Aguirre-Segura A, Miñano J, Lencina JL, Ortiz-Sánchez FJ, et al. Terrestrial arthropod fauna of arid areas of SE Spain: Diversity, biogeography, and conservation. J Arid Environ. 2011;75(12): 1321–1332.

[82] Vicente-Serrano SM, Cuadrat-Prats JM, Romo A. Aridity influence on vegetation patterns in the middle Ebro Valley (Spain): Evaluation by means of AVHRR images and climate interpolation techniques. J Arid Environ. 2006;66(2): 353–375.

[83] Moyano L. Estudio y seguimiento de la fauna de Orthoptera de un entorno natural sometido a un programa de restauración ecológica en el sur de la Península Ibérica. Doctoral Thesis. Universidad de Córdoba. 2014.

[84] David JF, Coulis M. Millipedes faced with drought: the life cycle of a Mediterranean population of *Ommatoiulus sabulosus* (Linnaeus) (Diplopoda, Julida, Julidae). Zookeys. 2015;510: 115–24. 10.3897/zookeys.510.8838 PMC4523768 26257538

[85] Gómez-Catasús J, Barrero A, Garza V, Traba J. Alondra ricotí –*Chersophilus duponti*. In: Salvador A, Morales MB, editors. Enciclopedia Virtual de los Vertebrados Españoles. Museo Nacional de Ciencias Naturales, Madrid; 2016. http://www.vertebradosibericos.org/

[86] Suárez F, Hervás I, Herranz J. Las alondras de España peninsular. Madrid: Dirección General para la Biodiversidad, Ministerio de Medio Ambiente y Medio Rural y Marino; 2009.

[87] Eraud C, Cadet E, Powolny T, Gaba S, Bretagnolle F, Bretagnolle V. Weed seeds, not grain, contribute to the diet of wintering skylarks in arable farmlands of Western France. Eur J Wildl Res. 2014;61(1): 151–161.

[88] Pérez-Granados C, López-Iborra GM, Garza V, Traba J. Breeding biology of the endangered Dupont’s Lark *Chersophilus duponti* in two separate Spanish shrub-steppes. Bird Study. 2017;64(3): 328–338.

[89] Mainwaring MC, Hartley IR. Causes and consequences of differential growth in birds. Adv Stud Behav. 2012;44: 225–277.

[90] Barrero A, Ovaskainen O, Traba J, Gómez-Catasús J. Co-occurrence patterns in a steppe bird community: insights into the role of dominance and competition. Oikos. 2023; e09780.

[91] Blondel J, Perret P, Anstett MC, Thebaud C. Evolution of sexual size dimorphism in birds: test of hypotheses using blue tits in contrasted Mediterranean habitats. J Evol Biol. 2002;15(3): 440–450.

[92] Alberdi A, Aizpurua O, Bohmann K, Gopalakrishnan S, Lynggaard C, Nielsen M, et al. Promises and pitfalls of using high-throughput sequencing for diet analysis. Mol Ecol Resour. 2019;19(2): 327–348. doi: 10.1111/1755-0998.12960 30358108

[93] Forsman AM, Hoenig BD, Gaspar SA, Fischer JD, Siegrist J, Fraser K. Evaluating the impacts of metabarcoding primer selection on DNA characterization of diet in an aerial insectivore, the Purple Martin. Ornithology. 2022;139(1): ukab075.

[94] Ducatez S, Sol D, Sayol F, Lefebvre L. Behavioural plasticity is associated with reduced extinction risk in birds. Nat Ecol Evol. 2020;4(6): 788–793. doi: 10.1038/s41559-020-1168-8 32251379

[95] Hallmann CA, Sorg M, Jongejans E, Siepel H, Hofland N, Schwan H, et al. More than 75 percent decline over 27 years in total flying insect biomass in protected areas. PLOS ONE. 2017;12(10): e0185809. doi: 10.1371/journal.pone.0185809 29045418 PMC5646769

[96] Wagner DL, Grames EM, Forister ML, Berenbaum MR, Stopak D. Insect decline in the Anthropocene: death by a thousand cuts. PNAS 2021;118(2): e2023989118. doi: 10.1073/pnas.2023989118 33431573 PMC7812858

[97] Fox R. The decline of moths in Great Britain: a review of possible causes. Insect Conserv Divers. 2013;6(1): 5–19.

[98] Reverter M, Gómez-Catasús J, Barrero A, Traba J. Crops modify habitat quality beyond their limits. Agric Ecosyst Environ. 2021;319: 107542.

[99] Grames EM, Montgomery GA, Youngflesh C, Tingley MW, Elphick CS. The effect of insect food availability on songbird reproductive success and chick body condition: Evidence from a systematic review and meta-analysis. Ecology Letters, 26(4), 658–673. Appl Ecol Environ Res. 2023;21(3): 1957–1978. doi: 10.1111/ele.14178 36798988

[100] Visser ME, Both C. Shifts in phenology due to global climate change: the need for a yardstick. Proc R Soc Lond B Biol Sci. 2005;272: 2561–2569. doi: 10.1098/rspb.2005.3356 16321776 PMC1559974

[101] Esbjerg P, Sigsgaard L. Phenology and pest status of Agrotis segetum in a changing climate. Crop Prot. 2014;62: 64–71.

[102] Irmler U. Seasonal shift in carabid phenology over a period of 18 years. Eur. J. Entomol. 2022;119: 183–190.

[103] Gómez-Catasús J. Habitat Quality, Wind Farms and Avian Population Trends in the Iberian Shrub-Steppe. Using a Threatened Passerine as a Model Species: The Dupont’s Lark *Chersophilus Duponti*. PhD thesis. Autonomous University of Madrid, Madrid; 2021.

[104] Gobindram N-E, Boughalmi A, Moulin CH, Meuret M, Bastianelli D, Araba A, et al. Feeding flocks on rangelands: insights into the local ecological knowledge of shepherds in Boulemane province (Morocco). Rangeland J. 2018;40(3): 207.

