# Supplementary figures and images for: Spatio-temporal variation of the endangered Dupont’s Lark diet across Iberia and Morocco
Source: PLoS One. 2024 Dec 11;19(12):e0301318. doi: 10.1371/journal.pone.0301318 (PMC11633968; doi:10.1371/journal.pone.0301318)

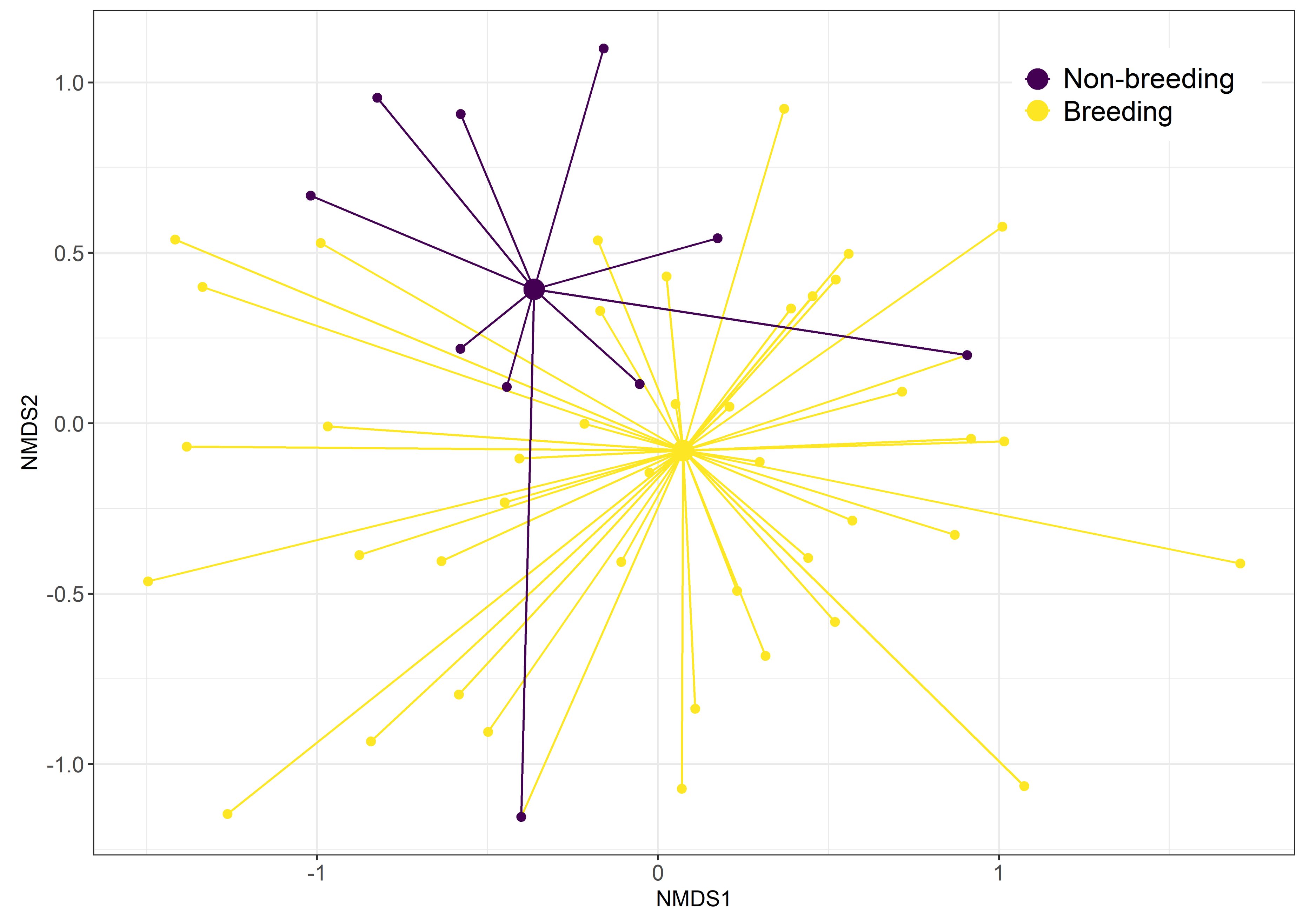

Supplement: S1 Fig — Smaller nodes represent individual birds with connecting lines joining the individual to the mean centroid (larger nodes) of its phenological period. (TIF) [file pone.0301318.s003.tif]

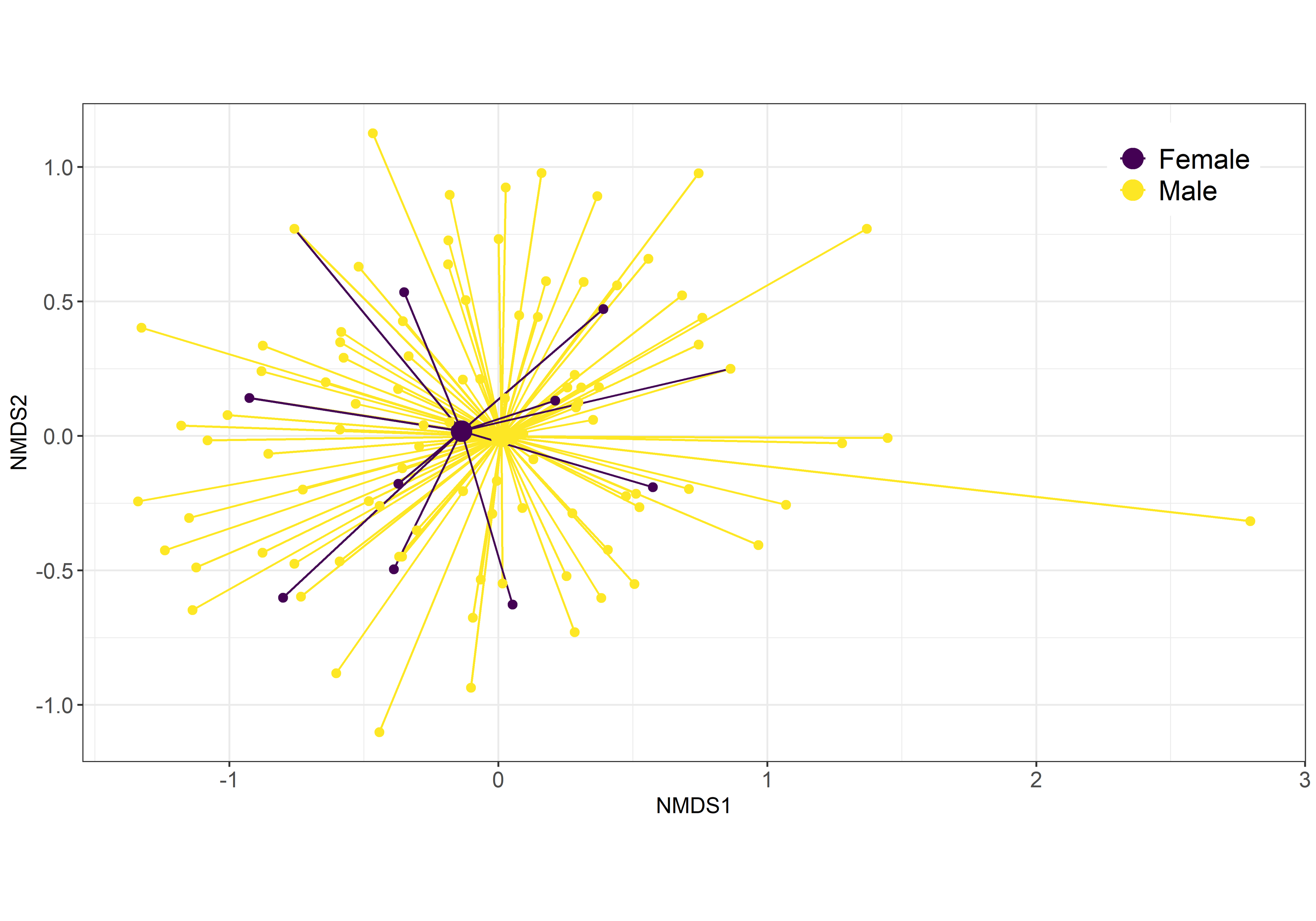

Supplement: S2 Fig — Smaller nodes represent individual birds with connecting lines joining the individual to the mean centroid (larger nodes) of its sex. (TIF) [file pone.0301318.s004.tif]
