# Supplementary material for: Spatio-temporal variation of the endangered Dupont’s Lark diet across Iberia and Morocco
Source: PLoS One. 2024 Dec 11;19(12):e0301318. doi: 10.1371/journal.pone.0301318 (PMC11633968; doi:10.1371/journal.pone.0301318)
Supplement: S1 Appendix — (DOCX) [file pone.0301318.s005.docx]

Spatio-temporal variation of the endangered Dupont’s Lark diet across Iberia and Morocco

Julia Zurdo^1,2*^, Daniel Bustillo-de la Rosa^1,2^, Adrián Barrero^1,2^, Julia Gómez-Catasús^1,2^, Margarita Reverter^1,2^, Cristian Pérez-Granados^3^, Jesús T. García^4^, Javier Viñuela^4^, Julio Domínguez^4^, Manuel B. Morales^1,2^, Juan Traba^1,2^

^1^ Terrestrial Ecology Group, Department of Ecology, Universidad Autónoma de Madrid (TEG-UAM), Madrid, Spain.

^2^ Centro de Investigación en Biodiversidad y Cambio Global, Universidad Autónoma de Madrid (CIBC-UAM), Madrid, Spain.

^3^ Ecology Department, Universidad de Alicante, Alicante, Spain.

^4^ Instituto de Investigación en Recursos Cinegéticos (IREC, CSIC-UCLM), Ciudad Real, Spain.

^5^ Pyrenean Institute of Ecology (IPE, CSIC), Jaca, Spain.

* Corresponding author

E-mail: julia.zurdo@uam.es (JZ)

**S1 Appendix. Location of Moroccan sampling points and sample size.**


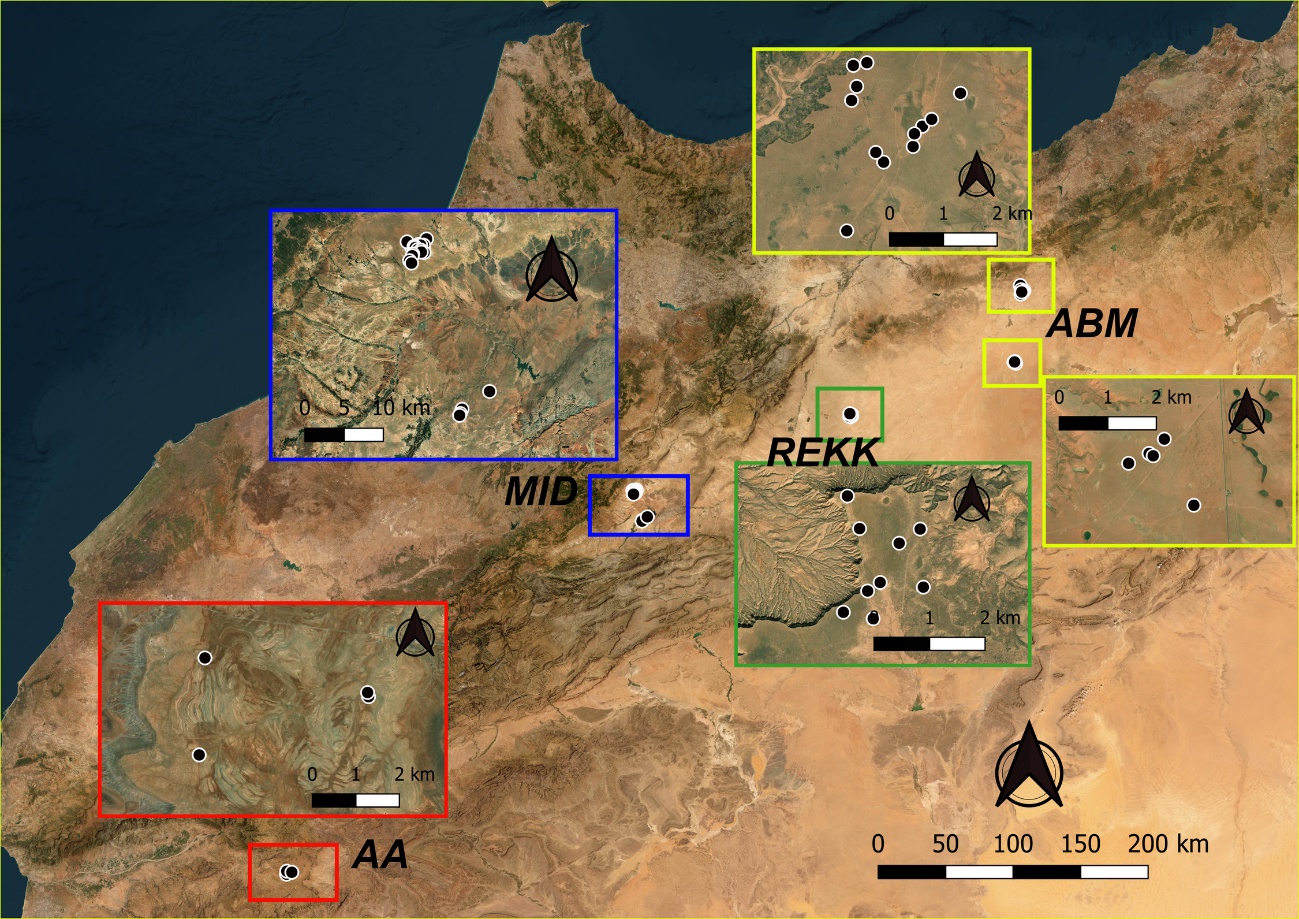


**Fig I. Detailed location of Moroccan sampling points** (black dots)**.** Fieldwork regions are indicated by the colored rectangles: Aïn Bni Mathar (ABM) in yellow, Plateau of Rekkam (REKK) in green, Midelt-Missour (MID) in blue, and Anti-Atlas (AA) in red.

**Table I. Number of Dupont’s Lark successful samples after molecular analysis across study regions of Morocco (colored rectangles in Fig I).**

| Moroccan region | *n* |
| --- | --- |
| Aïn Bni Mathar (ABM) | 13 |
| Plateau of Rekkam (REKK) | 6 |
| Midelt-Missour (MID) | 7 |
| Anti-Atlas (AA) | 2 |
