# Supplementary material for: Spatio-temporal variation of the endangered Dupont’s Lark diet across Iberia and Morocco
Source: PLoS One. 2024 Dec 11;19(12):e0301318. doi: 10.1371/journal.pone.0301318 (PMC11633968; doi:10.1371/journal.pone.0301318)
Supplement: S2 Appendix — (DOCX) [file pone.0301318.s006.docx]

Spatio-temporal variation of the endangered Dupont’s Lark diet across Iberia and Morocco

Julia Zurdo^1,2*^, Daniel Bustillo-de la Rosa^1,2^, Adrián Barrero^1,2^, Julia Gómez-Catasús^1,2^, Margarita Reverter^1,2^, Cristian Pérez-Granados^3^, Jesús T. García^4^, Javier Viñuela^4^, Julio Domínguez^4^, Manuel B. Morales^1,2^, Juan Traba^1,2^

^1^ Terrestrial Ecology Group, Department of Ecology, Universidad Autónoma de Madrid (TEG-UAM), Madrid, Spain.

^2^ Centro de Investigación en Biodiversidad y Cambio Global, Universidad Autónoma de Madrid (CIBC-UAM), Madrid, Spain.

^3^ Ecology Department, Universidad de Alicante, Alicante, Spain.

^4^ Instituto de Investigación en Recursos Cinegéticos (IREC, CSIC-UCLM), Ciudad Real, Spain.

^5^ Pyrenean Institute of Ecology (IPE, CSIC), Jaca, Spain.

* Corresponding author

E-mail: julia.zurdo@uam.es (JZ)

**S2 Appendix. Bioinformatic pipeline description.**

In this document we describe the pipeline followed for the bioinformatic analysis: Metabarcoding Joining Obitools & Linkage Networks In R (MJOLNIR).

This pipeline was followed for both molecular markers used in metabarcoding (18S and ZBJ), and was developed by Owen S. Wangensteen (UiT, The Arctic University of Norway).

R code:

# *Load MJOLNIR silently.*

suppressPackageStartupMessages(library(mjolnir))

# *Define number of cores to be used in parallel.*

cores <- 5

*# Input name for the final combined library (should be a 4-character name)*

lib <- "BIRD"

*## MJOLNIR pipeline ##*

*# FREYJA will do the paired-end alignment, demultiplexing & length filtering. This will be done for each sample file separately.*

*# Marker 18S:*

mjolnir2_FREYJA("", cores, Lmin=100, Lmax=600, lib, demultiplexed=T, R1_motif="_R1.", R2_motif="_R2.", primer_F="GGCCGTTCTTAGTTGGTGGA", primer_R="CCCGGACATCTAAGGGCATC")

*# Marker ZBJ:*

mjolnir2_FREYJA("", cores, Lmin=120, Lmax=230, lib, demultiplexed=T, R1_motif="_R1.", R2_motif="_R2.", primer_F="AGATATTGGAACWTTATATTTTATTTTTGG", primer_R="WACTAATCAATTWCCAAATCCTCC")

*# HELA will remove chimeric sequences in a sample-by-sample basis, will change identifiers of remaining unique sequences & will generate a table of their abundances in each sample & a fasta file with unique sequences and their total abundance for ODIN.*

mjolnir3_HELA(lib, cores)

*# ODIN will do the clustering & will generate a table with the abundances of each MOTU in each sample.*

*# Marker 18S:*

mjolnir4_ODIN(lib, cores, d=1, generate_ESV=FALSE)

*# Marker ZBJ:*

mjolnir4_ODIN(lib, cores, d=13, generate_ESV=FALSE)

*# THOR will assign the taxonomy to the representative sequence of each MOTU.*

*# Marker 18S:*

mjolnir5_THOR(lib, cores, tax_dir="~/taxo_NCBI", ref_db="ecoPCR_results_18S_final.fasta", taxo_db=" 18S_db", run_ecotag=T)

*# Marker ZBJ:*

mjolnir5_THOR(lib, cores, tax_dir="~/taxo_feb2022", ref_db="ecoPCR_results_COI_final.fasta", taxo_db="COI_db", run_ecotag=T)

*# FRIGGA will integrate the information of MOTU abundances and taxonomy assignment from ODIN & THOR in a single table.*

mjolnir6_FRIGGA(lib)

*# LOKI will remove the pseudogenes and will keep track of the taxonomic information of the removed MOTUs.*

mjolnir7_LOKI(lib, min_id=.84)

*# RAGNAROC will change the names of the samples to recover the original names and will remove unnecessary columns.*

mjolnir8_RAGNAROC(lib, "BIRD_metadata.tsv", "BIRD_final_dataset.csv", sort_MOTUs="taxonomy", remove_bacteria=T, remove_contamination=F, min_reads=3)
